# Identification of novel lipid metabolism-related biomarkers of aortic dissection by integrating single-cell RNA sequencing analysis and machine learning algorithms

**DOI:** 10.3389/fimmu.2025.1681989

**Published:** 2025-10-30

**Authors:** Zhechen Li, Yusong Deng, Fei Xiao, Jiashu Sun, Qixu Zhao, Zetong Zheng, Gang Li

**Affiliations:** 1 Beijing Luhe Hospital, Capital Medical University, Beijing, China; 2 Pediatric Cardiac Center, Beijing Anzhen Hospital, Capital Medical University, Beijing, China; 3 Beijing Institute of Heart Lung and Blood Vessel Diseases, Beijing, China; 4 Department of Anesthesiology, Beijing Anzhen Hospital, Capital Medical University, Beijing, China

**Keywords:** macrophage, lipid metabolism, aortic dissection, PLIN2, single-cellRNA sequencing

## Abstract

**Introduction:**

Aortic dissection (AD) is a lethal disease with increasing incidence and limited preventive options, characterized by aortic media degeneration and inflammatory cell infiltration. Dysregulation of lipid metabolism is increasingly recognized as a pathological characteristic of AD; however, the exact molecular regulators and critical genetic determinants involved remain unclear.

**Methods:**

This study employed an integrative approach combining single-cell RNA sequencing and machine learning to identify novel lipid metabolism-related biomarkers in aortic dissection. Single-cell RNA sequencing data from aortic dissection and control samples were processed to analyze lipid metabolism activity and identify differentially expressed genes. Machine learning algorithms and protein-protein interaction networks were then used to prioritize biomarkers, which were further validated through bulk RNA-seq analysis and immune infiltration studies and experiments using an Ang II-induced aortic dissection mouse model.. Functional characterization included cell-cell communication analysis and pseudo-time trajectory reconstruction to elucidate the roles of candidate genes in aortic dissection pathogenesis.

**Results:**

This multi-modal strategy identified PLIN2 and PLIN3 as key regulators of lipid metabolism in aortic dissection. Analysis revealed significant up-regulation of lipid metabolism in aortic dissection, with PLIN2 and PLIN3 emerging as central regulators. Single-cell profiling showed these genes were highly expressed in monocytic cells, correlating with enhanced inflammatory signaling (e.g., SPP1, GALECTIN). Machine learning and bulk RNA-seq validation confirmed their diagnostic potential. Pseudo-time analysis linked PLIN2 to early monocyte differentiation, while cell-cell communication studies implicated it in pro-inflammatory crosstalk with smooth muscle cells. The upregulation of PLIN2 and its specific expression in macrophages were further confirmed in an Ang II-induced aortic dissection mouse model. Molecular docking screened for potential therapeutic compounds that may target PLIN2, among which ketoconazole was identified.

**Discussion:**

These findings suggest that PLIN2/PLIN3 could be key mediators of metabolic dysregulation and immune activation in aortic dissection, highlighting their potential as diagnostic markers and therapeutic targets.

## 1 Introduction

Aortic dissection (AD), an aggressive cardiovascular disease with an exceedingly mortality rate (1). According to the Stanford classification, AD can be classified as Stanford type A (TAAD) or type B(TABD) (2). Aortic dissection arises from multiple pathological processes, including aberrant phenotypic switching and apoptosis of vascular smooth muscle cells, impaired extracellular matrix homeostasis, endothelial dysfunction, and inflammatory immune cell infiltration (3). TAAD constitutes approximately 67% of all AD cases, with mortality rates escalating rapidly at a rate of 2.6% per hour during the first 24 hours. Although significant advancements in the management of TAAD over the past decade, overall survival remains suboptimal, with postoperative mortality rates persisting at 10–35%, emphasizing the urgent need for early non-invasive intervention and preventive strategies (4, 5). Given these challenges, a deeper understanding of the molecular mechanisms driving AD is urgently needed to discover new therapeutic strategies and robust prognostic markers.

Lipids serve crucial functions in energy homeostasis, biological membrane architecture, and cellular signaling pathways. However, dysregulation of lipid metabolism contributes significantly to the pathogenesis of multiple disorders, including metabolic syndrome, type 2 diabetes mellitus, and cardiovascular diseases (6, 7). Similarly, in aortic dissection, clinical studies have identified significant dyslipidemia in AD patients, characterized by elevated serum total cholesterol and low-density lipoprotein (LDL) levels. Notably, oxidized LDL (OxLDL) upregulates TLR4 expression and activates the NF-κB pathway, thereby promoting inflammatory cell recruitment. These infiltrating immune cells exacerbate vascular wall injury through increased production of reactive oxygen species (ROS) and proteolytic enzymes (8, 9).

Although emerging evidence underscores the critical involvement of lipid metabolites in AD pathogenesis, the identification and functional validation of key regulatory genes remain a substantial challenge. Single-cell RNA sequencing (scRNA-seq) represents a powerful emerging technology that facilitates genomic profiling, cellular heterogeneity assessment, differential gene expression analysis, and cell-type identification at single-cell resolution (10, 11). When integrated with machine learning algorithms and complementary bioinformatics approaches, this methodology holds significant potential for discovering novel diagnostic biomarkers (12, 13).

This study represents the first demonstration of lipid metabolic heterogeneity at single-cell resolution in AD, revealing significant intercellular variations across distinct cell populations. Our study integrates single-cell and bulk transcriptomic data to systematically identify biomarkers associated with dysregulated lipid metabolism in AD pathogenesis. Moreover, using machine learning approaches, we refined these candidate genes and identified optimal feature genes linked to dysregulated lipid metabolism in AD. These findings suggest novel therapeutic targets and provide important mechanistic insights for future AD research and intervention strategies.

## 2 Materials and methods

### 2.1 Data acquisition and processing

We obtained the scRNA-seq data for AD from the GSE189795 (14), GSE254132 (15) and GSE222318 (16) from GEO database (http://www.ncbi.nlm.nih.gov/geo/). After merging all the datasets, we required 15 AD and 14 normal control samples. Furthermore,746 lipid metabolism-related genes were obtained from GSEA database (https://www.gsea-msigdb.org/gsea/index.jsp). To ensure high-quality single-cell data, we performed stringent quality control by filtering cells based on the following criteria: genes detected per cell (nFeature_RNA) between 200 and 5,000, total UMI counts (nCount_RNA) between 200 and 30,000, mitochondrial gene content (pMT) below 20%, and hemoglobin gene content (pHB) below 5%. After applying these thresholds, we retained 211235 high-qualified cells for subsequent analysis. To identify the most variable genes, the top 2000 were selected using the “FindVariableFeatures” function after applying linear regression-based “Log-normalization” (scale.factor = 10000) to scale and normalize the remaining cells. Following this, Principal Component Analysis (PCA) was employed to reduce the dimensionality of the single-cell RNA sequencing data. Then, we utilized the Harmony, a R package which is an algorithm for robust, scalable, and flexible multi-dataset integration (17), aiming to harmonize the data and remove batch-induced differences (group.by.vars = “orig.ident”).With pc.num=1:15 and a clustering resolution of 1, the “FindClusters” function partitioned cells into distinct groups based on reduced-dimensionality space. Besides, potential doublets were identified using the R package DoubletFinder (18), with parameters set at PCs = 1:15 and an expected doublet formation rate of 7%.The algorithm detected a subset of cells as doublets. Cell annotation was performed using well-established marker genes to classify distinct cell clusters into specific cell types. Besides, we chose the bulk RNAseq data GSE153434 (19) as the bulk RNA-seq data containing 10 AD samples and 10 normal control of human aortic tissue and GSE52093 (20) as the examining data containing 7 AD samples and 5 normal control. The details of all the data used in this research are provided in **Supplementary File 1**.

### 2.2 Single-cell level screening of potential lipid metabolism-related genes

In this study, we employed AUCell (21) to calculate pathway activity scores, evaluating lipid metabolic pathway engagement across all cells. To further validate the accuracy of the AUCell results, we also applied the AddModuleScore algorithm (22).Based on these scores, cells were stratified into high- and low-activity groups, followed by differential gene expression analysis with a criteria set at log2 fold change > 0 and adjusted pvalue<0.05 to identify up-regulated markers. Statistical comparisons were performed using the Wilcoxon rank-sum test. Significance levels were defined as follows: p < 0.05 (*), p< 0.01 (**), and p < 0.001 (***).Concurrently, Spearman correlation analysis was performed to select the top 128 genes most strongly associated with lipid metabolism. The intersection of these gene sets was then derived to pinpoint the most potential lipid metabolism-related genes.

### 2.3 Functional enrichment analysis

Functional enrichment analysis(pvalueCutoff = 0.05 and qvalueCutoff =0.05) was performed using Gene Ontology (GO) and Kyoto Encyclopedia of Genes and Genomes (KEGG) to characterize the biological pathways and protein functions associated with these genes. Metascape is an online platform for enrichment analysis that integrates functional enrichment, interactome analysis, and gene annotation, thereby enabling a comprehensive interpretation of gene functions (23).

### 2.4 Screening for optimal biomarkers

To systematically identify lipid metabolism related biomarkers, we implemented a multi-platform validation strategy. First, three machine learning approaches - LASSO regression (24), Random Forest(RF) (25) (2000 trees with Gini importance scoring), Boruta algorithm (26) and Support Vector Machine(SVM) -were employed for robust feature selection.

Specifically, prior to LASSO regression analysis, the randomcoloR package in R was utilized to generate 40 distinct colors, with the random seed set at set.seed (1). The LASSO model was then executed with set.seed (11) and an alpha value of 1. For the Random Forest model, the random seed was fixed at set.seed (3). The Boruta algorithm was implemented with set.seed (1), along with parameters doTrace = 2 and maxRuns = 500. The SVM model was configured using 5-fold cross-validation (nfold = 5). Detailed parameter settings for SVM are provided in the **Supplementary File 2**.

Concurrently, potential candidates were subjected to protein-protein interaction(PPI) analysis through STRING database (https://cn.string-db.org/) followed by Cytoscape (27) visualization and MCODE (28) clustering. The intersection of genes identified by all five independent methods (LASSO, Random Forest, Boruta, SVM and PPI cluster cores) was subsequently derived, yielding a high-confidence set of biomarkers. This consensus approach minimizes methodological bias while enhancing biological relevance through multi-algorithm cross-validation and systematic parameter configuration. Finally, the expression patterns of these biomarkers were visualized using violin plots, while their diagnostic potential was rigorously evaluated through receiver operating characteristic (ROC) curve analysis, including calculation of the area under the curve (AUC) in both training and examining data.

### 2.5 Immune infiltration

We investigated the relationship between the biomarkers and the immune cells by first profiling immune infiltration patterns using marker genes curated from the Gene Set Enrichment Analysis database(GSEA database, https://www.gsea-msigdb.org/). Spearman correlation analysis revealed significant associations between each biomarkers and specific immune cell subtypes (29). Subsequently, single-gene GSEA was performed on the GSE153434 dataset by: 1. ranking all genes based on their correlation coefficients with each biomarkers, and 2. analyzing these ranked lists using a R package, ClusterProfiler, with the ‘c5.go.v2023.2.Hs.symbols.gmt’ gene set from the Molecular Signatures Database (MSigDB, https://www.gsea-msigdb.org/gsea/msigdb/index.jsp). Finally, we visualized the results through bubble plots summarizing significantly enriched pathways, providing a comprehensive understanding of the function of two up-regulated biomarkers.

### 2.6 Cell communication

Based on median expression levels of PLIN2, monocytic cells were stratified into high- and low-expression groups. To further understand the cell communication difference between the high-expression group and low-expression group, we performed cell-cell communication analysis. This analysis was conducted using the CellChat R package, which infers intercellular communication by integrating known ligand-receptor interactions and gene expression profiles, enabling the identification of signaling pathways and communication patterns between different cell types (30). By comparing the communication networks between high- and low-expression groups, we identified significant differences in cell-cell interactions, providing insights into the underlying signaling pathways involved in disease progression.

### 2.7 CytoTRACE and pseudo-time analysis

To assess the differentiation potential of monocytic cell populations, we utilized CytoTRACE for quantitative evaluation of developmental progression across distinct monocytic subsets. For trajectory reconstruction, cells were systematically classified into two distinct states based on their gene expression profiles using Monocle pseudo-temporal ordering analysis. To delineate temporally regulated genes during monocytic differentiation, we conducted comprehensive differential expression analysis along the pseudotime continuum (differentialGeneTest). We used the “plot_genes_in_pseudotime” to show the dynamic expression patterns of the targeted genes. Lastly, we performed differential gene expression analysis (min.pct = 0.01,logfc.threshold = 0.01,test.use=“wilcox”) and GSEA(pvalueCutoff = 0.05) between PLIN2 labeled monocytic cells.

### 2.8 Molecular docking

We utilized the online databases Drug-Gene Interaction Database (31) (DGIdb, https://dgidb.org/) and Drug Signatures Database (32) (DSigDB, https://dsigdb.tanlab.org/) to predict potential drug targets for the biomarker. Four candidate drugs were selected for molecular docking. First, the molecular structures of the drugs were downloaded from PubChem (https://pubchem.ncbi.nlm.nih.gov/), and their most stable conformations were generated using Chem3D. Subsequently, the predicted protein structure of the target was obtained from the AlphaFold Protein Structure Database (33) (https://alphafold.com). Molecular docking was then performed using AutoDock Vina (34).The specific parameters for the docking simulation, including the center coordinates (X, Y, Z) and dimensions of the docking box, are provided in **Supplementary File 3**, followed by visualization of the interactions using the Protein-Ligand Interaction Profiler (35) (PLIP, https://plip-tool.biotec.tu-dresden.de/plip-web/plip/index) and PyMOL.

### 2.9 Animal model

Aortic dissection (AD) was induced in ApoE⁻/⁻ mice using a well-established model of Angiotensin II (Ang II) infusion (36, 37). Ten mice were randomly allocated into an experimental group (n=10) and a control group (n=10). Mice in the experimental group received a continuous subcutaneous infusion of Ang II (1000 ng/kg/min) via osmotic minipumps for 4 weeks, a protocol demonstrated to reliably induce aortic pathologies including dissection and aneurysm in hyperlipidemic mice (36). The control group received saline. Following the infusion period, 10 mice in the Ang II group developed aortic dissection, confirmed by gross examination, whereas no dissections were observed in the control group. Our results are consistent with the established efficacy of this model in promoting AD in ApoE⁻/⁻ mice.

This study was approved by the Ethics Committee of Beijing Anzhen Hospital, Capital Medical University (Approval No. AZ2025LA015). All experimental procedures were conducted in strict accordance with the ethical principles for the care and use of laboratory animals.

### 2.10 Western blotting

Proteins were extracted from aortic tissues and quantified using a BCA protein assay kit (Beyotime Biotechnology, Cat# P0010). The samples were then mixed with loading buffer, denatured by heating at 95 °C for 5 minutes, and separated by 10% SDS-PAGE. Subsequently, the separated proteins were transferred onto a PVDF membrane. After blocking with 5% BSA, the membrane was incubated overnight at 4 °C with the following primary antibodies: anti-Plin2 (1:1,000, Proteintech, Cat# 15294-1-AP, rabbit polyclonal) and anti-GAPDH (1:50,000, Proteintech, Cat# 60004-1-Ig, mouse monoclonal). Following primary antibody incubation, the membrane was incubated with a mixture of fluorescent dye-conjugated secondary antibodies: IRDye 800CW goat anti-rabbit IgG (1:20,000, Bioss, Cat# bs-40295G-IRDye800CW) and IRDye 800CW goat anti-mouse IgG (1:20,000, Bioss, Cat# bs-40296G-IRDye800CW). Protein bands were finally visualized and quantified using an Odyssey infrared imaging system.

### 2.11 qRT-PCR

Total RNA was extracted from aortic tissue and reverse-transcribed into cDNA using the Evo M-MLV RT Premix Kit (Accurate Biology, Cat# AG11728). Subsequently, quantitative PCR (qPCR) was performed with the SYBR Green Pro Taq HS Premixed qPCR Kit (Accurate Biology, Cat# AG11701). The primer sequences used were as follows:

Plin2 (forward: 5′-TCCTTTCTGTTTGCACGTCCT-3′, reverse: 5′-CTCTCATCACCACGCTCTGT-3′) and the reference gene Gapdh (forward: 5′-ACCCTTAAGAGGGATGCTGC-3′, reverse: 5′-CCCAATACGGCCAAATCCGT-3′).

The qPCR cycling protocol consisted of an initial denaturation at 95 °C for 30 s, followed by 40 cycles of 95 °C for 5 s and 60 °C for 30 s. All reactions were carried out on an Applied Biosystems 7500 Real-Time PCR System.

### 2.12 Immunofluorescence

Mouse arterial tissue slices were obtained from samples embedded in paraffin. For immunofluorescence staining according to the manufacturer’s instructions, the samples were incubated overnight at 4 °C, and stained with PLIN2 polyclonal antibody (1:1000; GB113109; Wuhan Servicebio Technology Co., Ltd., Wuhan, China) and a CD68 polyclonal secondary antibody (1::1000; GB115593; Wuhan Servicebio Technology Co., Ltd., Wuhan, China). Slides were counterstained with DAPI. Finally, images were acquired using a fluorescence microscope (Rueil-Malmaison).

## 3 Results

### 3.1 The scRNA-seq profiling of AD

**Figure 1:**
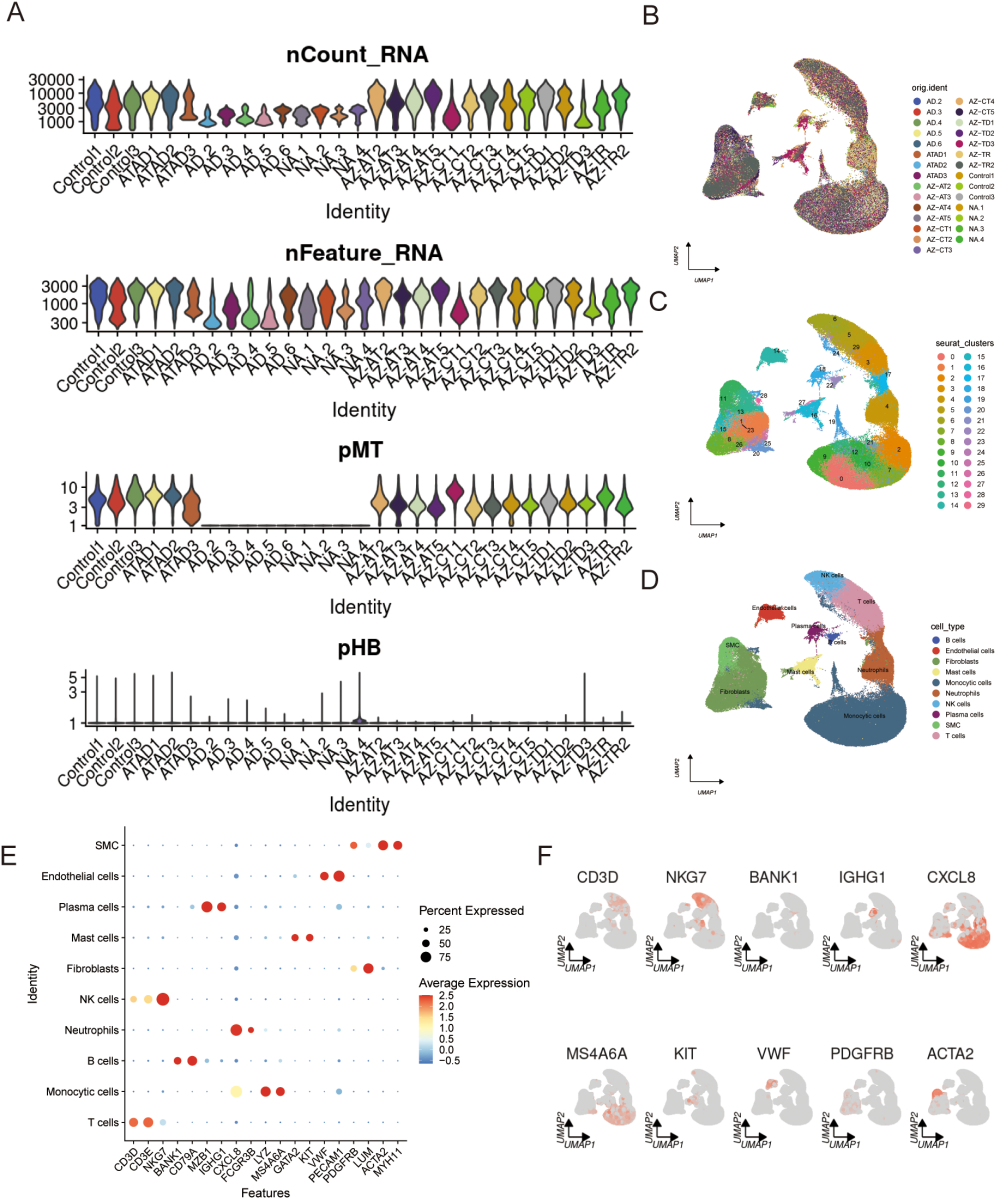
Screening of single-cell data. **(A)** Quality control of scRNA-seq data. **(B)** The UMAP results showed that the cell distribution was consistent across all samples, with minimal batch effects. **(C)** UMAP clustering divided all cells into 30 well-defined clusters. **(D, E)** The dataset was annotated into 10 cell populations based on established marker gene expression. **(F)** The expression pattern of the marker genes.

Quality control played a critical role in ensuring data reliability for downstream analysis. After rigorous assessment (**Figure 1A**), 29 high-quality samples (15 from the AD group and 14 from the normal control group) were selected for further investigation. The samples were sourced from three distinct datasets as follows: Control1–3 and ATAD1–3 were obtained from GSE254132; AD.2–6 and NA.1–4 were derived from GSE189795; and the remaining samples originated from GSE222318. To minimize technical variability, we performed batch effect correction using Harmony. This effectively harmonized the data distribution and eliminated batch-related differences, as assessed by the clear reduction in batch effect shown in **Supplementary Figure 2A**. The cells are well-integrated across batches, with no discernible batch-specific clustering, indicating the successful removal of technical variation (**Figure 1B**). Subsequent clustering analysis categorized all cells into 30 distinct clusters (**Figure 1C**).

Potential doublets were identified using the R package DoubletFinder. We first determined the optimal pK value, which yielded the highest cross-validation mean area under the curve (AUC), indicating the best model performance (**Supplementary Figure 1A**). The algorithm subsequently computed a doublet score for each cell and flagged a subset as putative doublets. As expected, these algorithm-identified doublets generally exhibited higher gene counts than singlet cells (**Supplementary Figure 1B**). However, visualization in the UMAP embedding revealed a heterogeneous distribution pattern: while some putative doublets formed isolated clusters, a substantial proportion were intermingled with transcriptically defined singlet populations (**Supplementary Figure 1C**). Given that actively metabolizing cells—a key focus of our study—can share high gene detection rates with technical doublets and thus be prone to misclassification, we adopted a conservative strategy. To avoid the inadvertent loss of these biologically relevant cells, all putative doublets were retained in downstream analyses.

Using established cell markers, we identified 10 major cell types, including B cells, endothelial cells, fibroblasts, and mast cells, among others (**Figure 1D**). The specificity of these cell populations was further confirmed by examining the expression patterns of key markers (**Figures 1E, F**). To rule out the possibility that batch integration introduced spurious findings, we independently processed and analyzed the three single-cell datasets. The key results were consistently reproduced in each individual data (**Supplementary Figures 3A–C**), confirming the robustness of our findings.

Comparative analysis revealed notable differences in cell type distribution between the two groups (**Supplementary Figure 4A**). Specifically, monocytic cells and smooth muscle cells were more abundant in the AD group, whereas plasma cells and fibroblasts showed reduced proportions (**Supplementary Figure 4B**). A detailed breakdown of cell type distribution across individual samples is provided (**Supplementary Figure 4C**).

### 3.2 Lipid metabolism in scRNA-seq data and enrichment analysis

**Figure 2:**
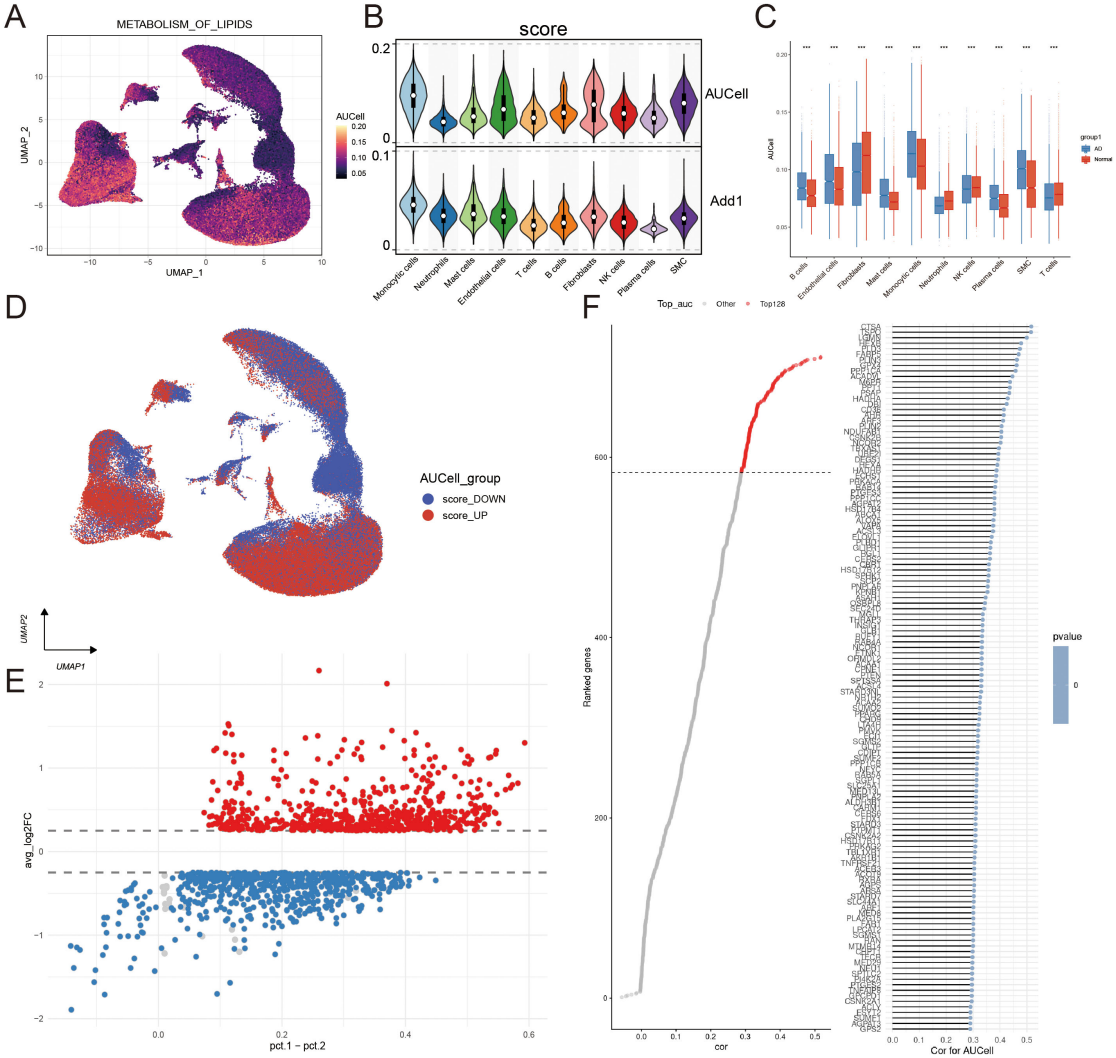
Analysis of lipid metabolism level at single-cell resolution in AD. **(A)** UMAP visualization revealed the global lipid metabolic profile across cell populations. **(B)** The box plot displays the lipid metabolism scores for different cell types, calculated using the AUCell and AddModuleScore algorithms. **(C)** Box plots illustrate the differential lipid metabolism levels across distinct cell populations between AD patients and normal controls.The blue and red colors represent the AD group and the normal control group, respectively.**(D)** UMAP visualization delineates cell clusters segregated into score-DOWN and score-UP lipid metabolism expression groups.**(E)** The volcanic plot shows the result of DEGs of lipid metabolism. **(F)** Top 128 genes selection was based on correlation analysis with lipid metabolic activity, retaining only statistically significant associations. The left panel shows the correlation coefficients for the top 128 genes, with a corresponding list of gene names, p-values, and correlation coefficients provided in the right panel.

Emerging evidence indicates that dysregulated lipid metabolism plays a critical role in the development of aortic dissection (9). In our study, we utilized the AUCell and AddModuleScore to evaluate lipid metabolism in scRNA-seq level(**Figure 2A**). The overall level of lipid metabolism was up-regulated compared to normal control. It is clear that there is great difference of the lipid metabolism level in different cell types. Among them, the UAMP plot clearly showed that these lipid metabolism-related genes were predominantly expressed in monocytic cells and fibroblasts(**Figure 2B**). To be specific, a comparative analysis of the normal group and AD group indicated that lipid metabolism-related genes were more active in monocytic cells and smooth muscle cells mainly while relatively suppressed in fibroblasts, T cells and NK cells, ect.(**Figure 2C**). Subsequently, by using the average expression score, all cells were classified into score_up group(above the mean) and score_down group(below the mean). The score_up group contains monocytic cells and fibroblasts predominantly(**Figure 2D**). Then we required 678 up-regulated differentially genes among the two groups(**Figure 2E**). To identify the genes most closely related with lipid metabolism, we used a spearman correlation analysis and identified 128 the most related to lipid metabolism (**Figure 2F**). To validate the robustness of our findings against potential integration artifacts, we repeated the lipid metabolism analysis on each dataset separately. The key results were consistently recapitulated: a significant dysfunction of lipid metabolic activity was confirmed in all three cohorts, with macrophages and fibroblasts consistently showing the highest enrichment scores (**Supplementary Figures 5A–C**).The AUCell score of all cells in AD group, the full lists of these 678 up-regulated genes and Top128 genes are provided in **Supplementary File 4**-**6** respectively.

### 3.3 Multiple enrichment analysis

**Figure 3:**
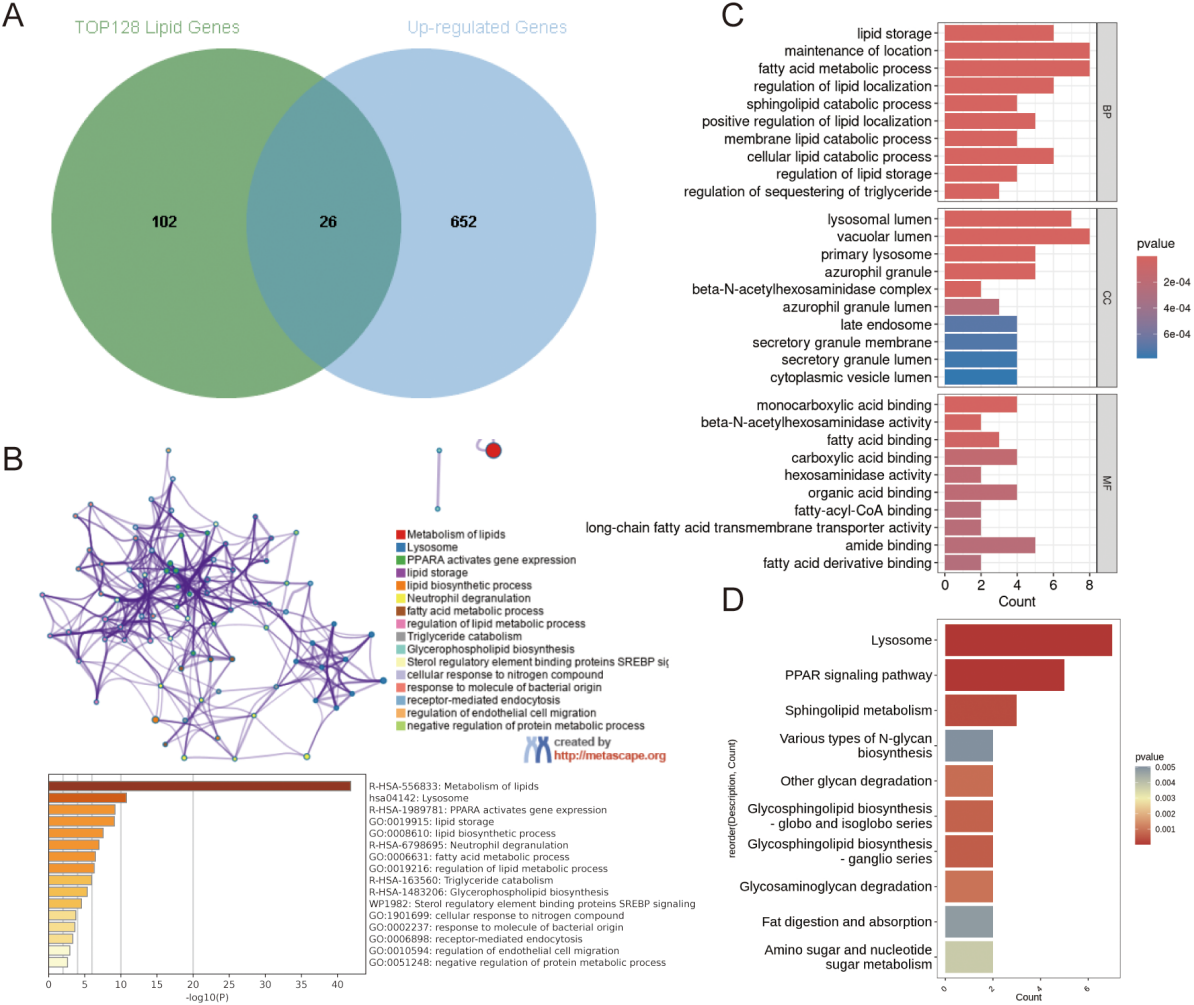
Multiple enrichment analysis. **(A)** The Venn diagram illustrates 26 genes overlapped by Top 128 lipid metabolism related genes and the up-regulated genes in AD group.**(B)** The metascape enrichment analysis shows the biological function of the 26 genes by network graph and bar chart. **(C, D)** GOBP,GOCC,GOMF and KEGG enrichment analysis of 26 up-regulated genes.

The intersection of these genes and up-regulated differentially expressed genes yielded 26 up-regulated genes highly associated with lipid metabolism (**Figure 3A**). The full process of the Venn diagram are offered in **Supplementary File 7**. To infer the biological functions of the 26 identified genes, we performed enrichment analysis using Metascape. The results revealed their most significant associations with lipid-related processes, including lipid metabolism, lysosomal function, the PPARA signaling pathway, and lipid storage, indicating a strong link between our gene set and lipid metabolic pathways (**Figure 3B**). Subsequent KEGG and GO enrichment analyses of these 26 genes identified distinct functional patterns. Gene Ontology analysis revealed three key functional clusters: molecular functions were predominantly associated with amide binding; biological processes showed enrichment in cellular localization maintenance, fatty acid metabolism, and lipid storage regulation; while cellular components were primarily localized to vacuolar and lysosomal luminal spaces (**Figure 3C**). KEGG pathway analysis demonstrated significant enrichment in lysosome-related pathways and PPAR signaling (**Figure 3D**). These findings collectively suggest the candidate genes’ potential roles in lipid metabolic regulation and lysosome-mediated cellular processes.

### 3.4 Identification of the biomarkers in bulk-RNA data

**Figure 4:**
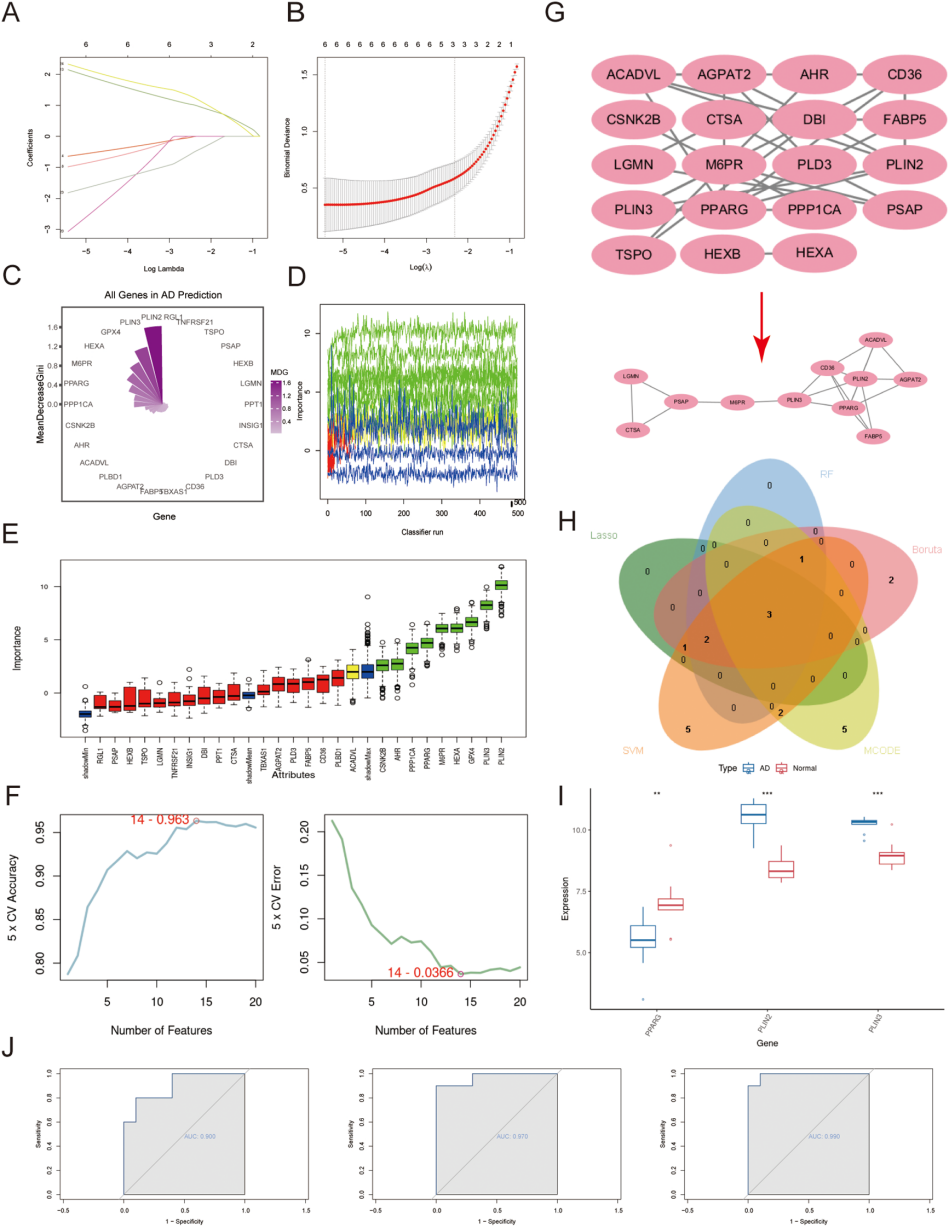
Screening for biomarkers based on bulk-RNA data. The machine learning containing the LASSO **(A, B)** algorithm,the Random Forest algorithm **(C)**, the Boruta algorithm **(D, E)**, the SVM algorithm **(F)** and PPI result visualized by Cytoscape as well as MCODE **(G)** determined the potential biomarkers. **(H)** The Venn diagram illustrates 3 biomarkers overlapped by the above-mentioned methods’ results. **(I)** Box plots demonstrate the differential expression patterns of three biomarkers between AD patients and normal controls, revealing a significant downregulation of PPARG and concurrent upregulation of PLIN2 and PLIN3 in the AD group. **(J)** ROC curves of the three biomarkers are displayed from left to right: PPARG (AUC = 0.900), PLIN3 (AUC = 0.970), and PLIN2 (AUC = 0.990).

To identify the most promising biomarkers, we employed a multi-step computational strategy. Four distinct machine learning algorithms—LASSO regression, Random Forest, Boruta, and SVM—were applied to the bulk RNA-seq data to select features most predictive of the phenotypic groups (**Figures 4A-F**). The application of LASSO, Random Forest, Boruta, and SVM yielded gene sets of 6, 6, 9, and 14 genes, respectively. The result of four machine learning algorithms are offered in **Supplementary File 8**-**11**. Concurrently, PPI network analysis followed by MCODE module detection identified 11 key hub genes (**Figure 4G**). By intersecting the results from all methods, we identified three key biomarkers:PLIN2,PLIN3 and PPARG (**Figure 4H**). Notably, PLIN2 and PLIN3 exhibited significantly increased expression, whereas PPARG was markedly down-regulated (**Figure 4I**). ROC curve analysis further confirmed their strong diagnostic potential, with AUC values of 0.900 (PPARG), 0.970 (PLIN3), and 0.990 (PLIN2) (**Figure 4J**) in training data.

### 3.5 Single gene set enrichment analysis and immune infiltration

**Figure 5:**
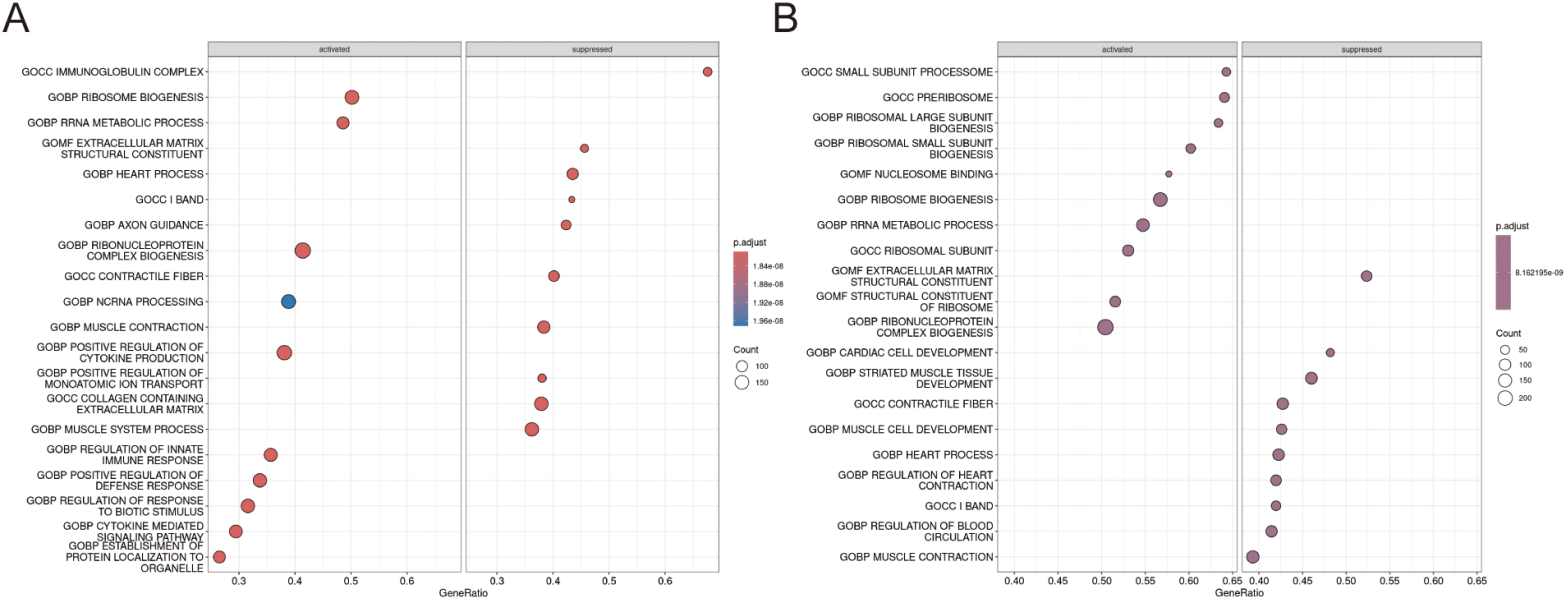
Elucidating the biological mechanisms of up-regulated biomarkers. **(A, B)** The GSEA results of PLIN2 and PLIN3.Each panel is composed of ‘Activated’ and ‘Suppressed’ sections, representing the activation status of the relevant pathway. The plot also displays the gene enrichment count and p-value for each pathway.

GSEA of the GSE15434 dataset revealed distinct pathway associations for the up-regulated biomarkers. PLIN2 activation showed enrichment in ribonucleoprotein complex biogenesis and ncRNA processing pathways, while its suppression was linked to immunoglobulin complex-related processes (**Figure 5A**). Similarly, PLIN3 activation correlated with ribosome biogenesis and nucleosome binding, whereas suppression was associated with muscle and heart development pathways (**Figure 5B**). The output of single GSEA for PLIN2 and PLIN3 are offered in **Supplementary File 12**, **13**. Immune infiltration analysis demonstrated significant correlations between these genes and immune cell populations: PPARG positively correlated with NKT cells and effector memory T cells but negatively with CD56dim NKT cells; PLIN2 associated with dendritic cells, neutrophils, and NK cells; and PLIN3 showed connections with central memory CD4+ T cells but negative correlations with NK cells and neutrophils (**Supplementary Figure 6A**). These findings suggest these genes may influence aortic dissection pathogenesis through their roles in metabolic pathways and immune cell regulation.

### 3.6 Validation PLIN2 and PLIN3 in in bulk and scRNA-seq data

**Figure 6:**
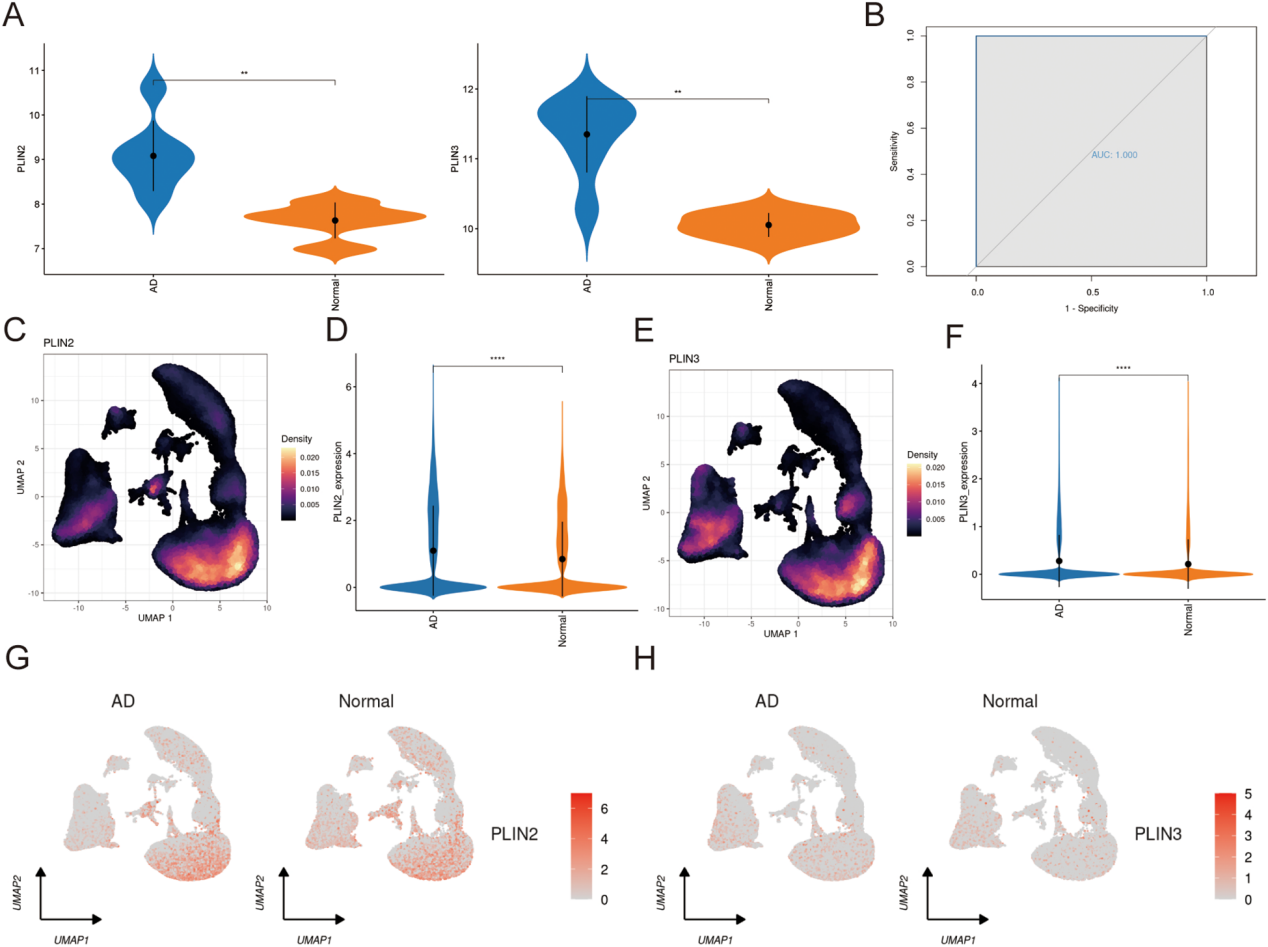
Validation PLIN2 and PLIN3 in bulk and single cell RNA-seq data. **(A)** The expression pattern of PLIN3 and PINL2 in GSE52093. **(B)** ROC curves of PLIN2 and PLIN3 in GSE52093(AUC = 1.00). **(C, E)** The distribution of PLIN3 and PLIN2 expression across different cells types. **(D, F)** The violin plots show PLIN2 and PLIN3 were significantly upregulated at the single-cell level in the AD group. **(G, H)** The UMAP visualization displays the expression levels of PLIN2 and PLIN3 in both the AD and normal groups.

In examining data, analysis revealed a significant upregulation of PLIN2 and PLIN3 in the AD group compared to the normal controls. Both genes achieved an AUC of 1.00, demonstrating their exceptional diagnostic potential for the disease (**Figures 6A, B**). Notably, these genes were primarily expressed in monocytic cells and significant up-regulated in AD progression in scRNA-seq data (**Figures 6B–D**). To further substantiate our findings, we validated the expression patterns of PLIN2 and PLIN3 in each of the three individual, non-integrated single-cell datasets. Reassuringly, the overall distribution and upregulation trend of both genes were consistently recapitulated across all separate cohorts, aligning greatly with the observations from the merged dataset(**Supplementary Figures 7A–D**).

### 3.7 Intercellular communication

**Figure 7:**
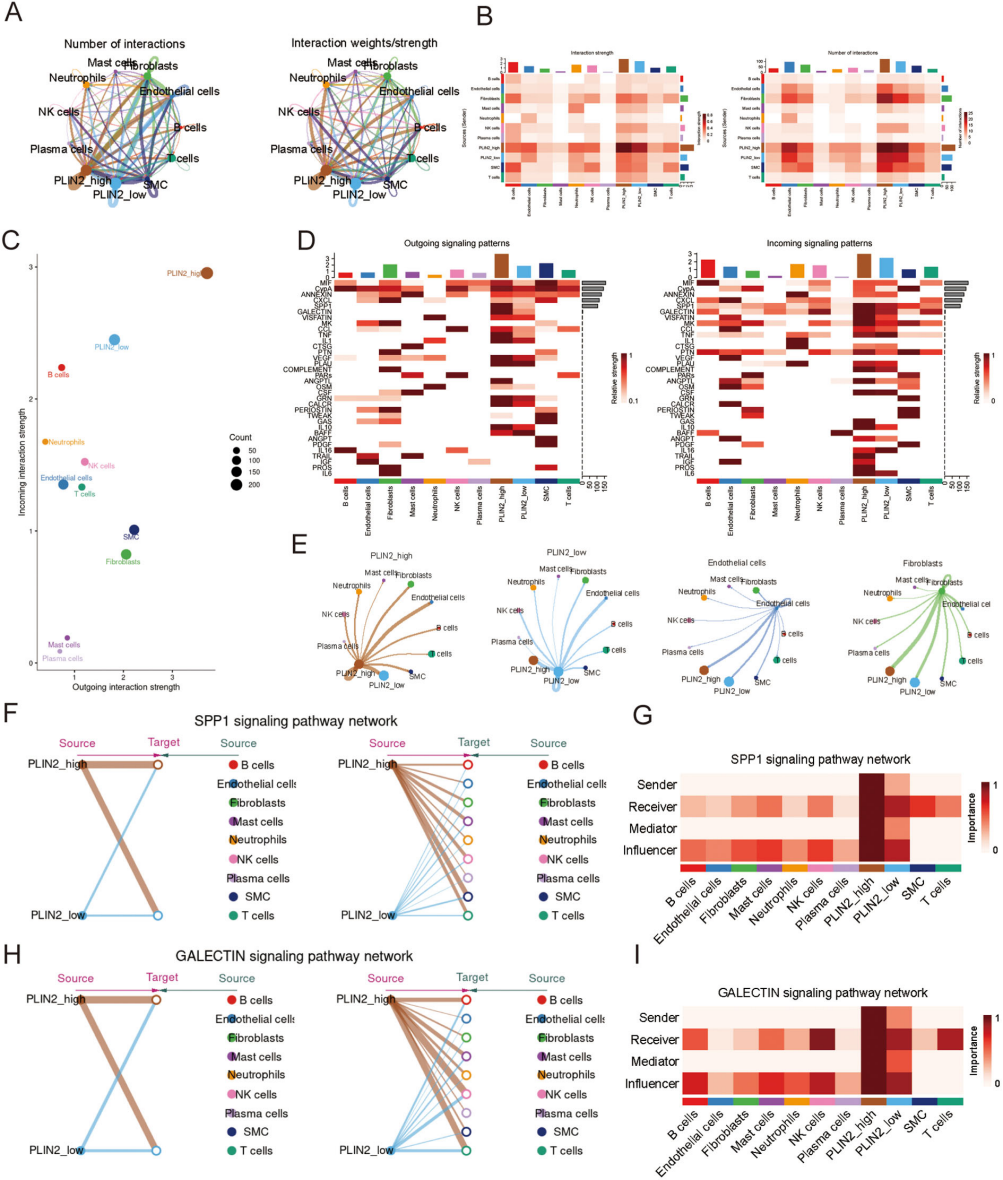
Celluar communication pattern in AD group. **(A–C)** The chord diagrams,heatmap and scatter plot exhibit the number and strength of interaction between PLIN2-high and PLIN2-low groups and other cell types. **(D)** The heatmaps show the outgoing and incoming interaction pattern among different cell types. **(E)** The chord diagrams illustrate the interaction of PLIN2_high,Plin2_low,endothelial cells and fibroblats. **(F–I)** The hierarchy plot and the heatmap respectively illustrate the hierarchical relationship of the SPP1 and GALECTIN pathways and the roles played by different cell types.

Based on the median expression level of PLIN2 in monocytic cells, these cells were stratified into PLIN2-high and PLIN2-low subgroups. The analysis included 27,312 and 30,045 monocytic cells in the PLIN2-high and PLIN2-low groups, respectively. Further investigation revealed that PLIN2-high monocytic cells exhibited significantly stronger cell-cell communication with other cell types, particularly endothelial cells and fibroblasts (**Figures 7A–C**). Both incoming and outgoing interaction intensities were markedly higher in the PLIN2-high group compared to the PLIN2-low group (**Figure 7D**). The PLIN2-high group exhibited more active communication with both fibroblasts and endothelial cells than the PLIN2-low group. For both fibroblast and endothelial cells, the strongest interactions originated from the PLIN2-high group(**Figure 7E**). Key signaling pathways involved in these interactions included IL10, SPP1, ANGPTL, GALECTIN, and TNF signal. In the SPP1 signaling network, the PLIN2-high population was identified as the primary source. In contrast, the PLIN2-low group, SMC, and T cells were the main receivers. Furthermore, the PLIN2-high group also served as the most prominent mediator and influencer, underscoring its critical role in orchestrating SPP1-mediated communication (**Figures 7F, G**). Similarly, for the GALECTIN pathway, the PLIN2-high group was once again the most active. It primarily sent signals to cells such as NK cells and T cells, establishing itself as a key player in this network (**Figures 7H, I**).

### 3.8 CytoTRACE and trajectory analysis

**Figure 8:**
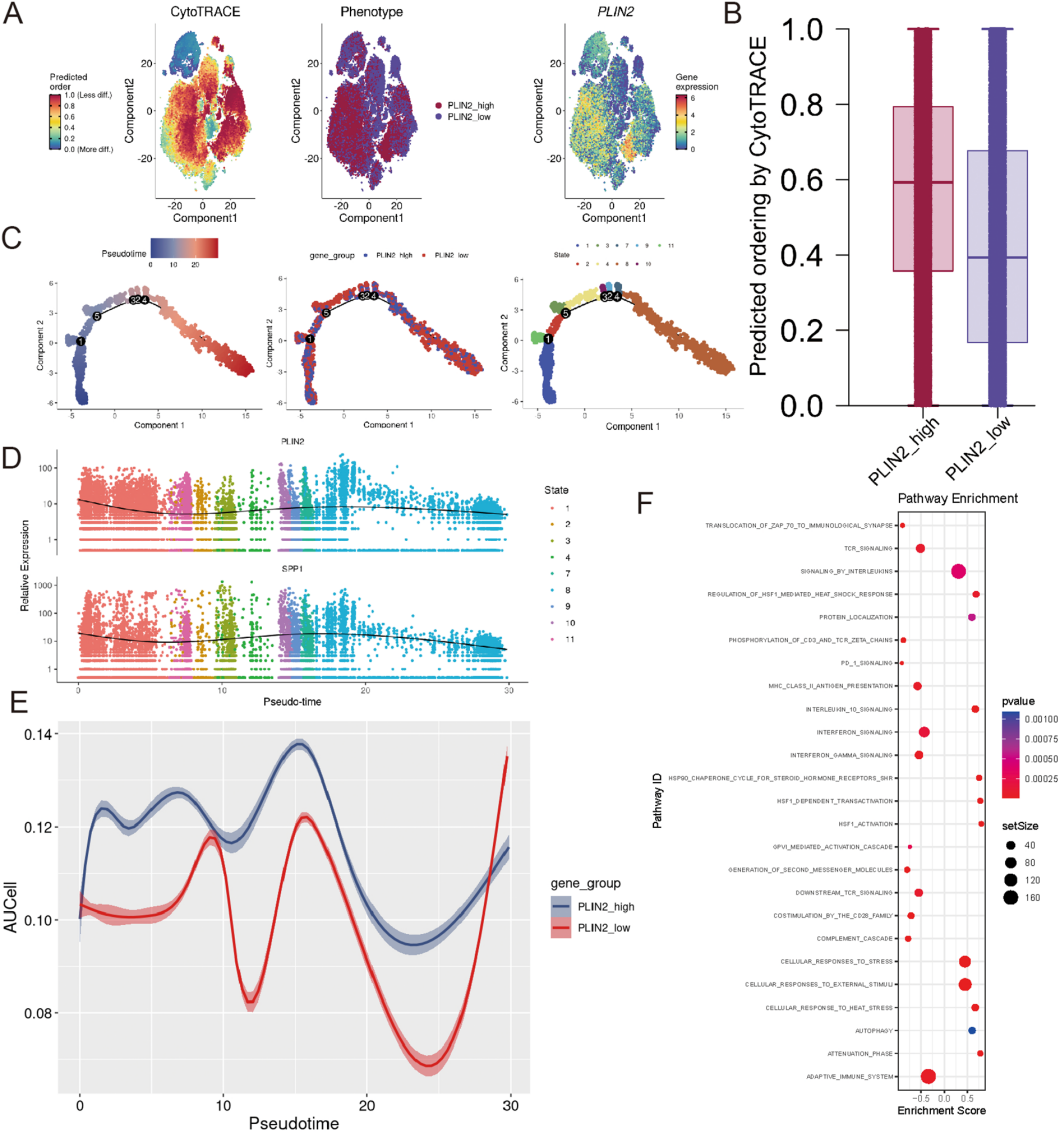
CytoTRACE and trajectory analysis of PLIN2 labeled monocytic cells in AD group **(A)** CytoTrace,Phenotype and PLIN2 expression pattern of tSNE diagram. **(B)** Bar graph shows the differentiation status of PLIN2 labeled monocytic cells. **(C)** Pseudo-time analysis predicts the differentiation trajectory of PLIN2-labeled monocytic cells. **(D)** Pseudotime analysis revealed the dynamic expression pattern of PLIN2 and SPP1. **(E)** The dynamic expression pattern of lipid metabolism between PLIN2_high and PLIN2_low group **(F)** GSEA of up-regulated DEGs between PLIN2-high and PLIN2-low groups.

CytoTRACE was employed to define the development order of the monocytic cells, revealing that PLIN2-high monocytic cells resided in the early stages of development with better differentiation potential (**Figures 8A, B**). Besides, we found that as pseudo-time progressed, monocytic cells differentiated from left to right with 11 different cell states (**Figure 8C**). The differential GeneTest identified the genes with the most significant expression changes along the pseudo-time trajectory. Among these most dynamically altered genes, SPP1 exhibited an expression pattern similar to PLIN2 across pseudotime. The parallel expression dynamics (initial downregulation-transient upregulation-secondary decline) imply possible functional crosstalk between SPP1 and PLIN2 (**Figure 8D**). We next performed pseudotime analysis and evaluated its relationship with lipid metabolism activity (as measured by AUCell scores) using ggplot2. The results revealed substantial fluctuation in lipid metabolic activity along the pseudotime trajectory. Notably, the PLIN2-high group exhibited consistently elevated scores compared to the PLIN2-low group throughout this process, underscoring the critical role of PLIN2 in lipid metabolism (**Figure 8E**). Lastly, we performed differential gene expression analysis and GSEA between PLIN2-high and PLIN2-low groups. The PLIN2-high group showed significant enrichment in several pathways including IL-10 signaling, cellular responses to external stimuli, cellular responses to stress, and autophagy. These pathways may be functionally associated with PLIN2’s role in AD pathogenesis (**Figure 8F**). The list of differential gene expression are provided in **Supplementary File 14**.

### 3.9 Binding of PLIN2 to potential drugs

**Figure 9:**
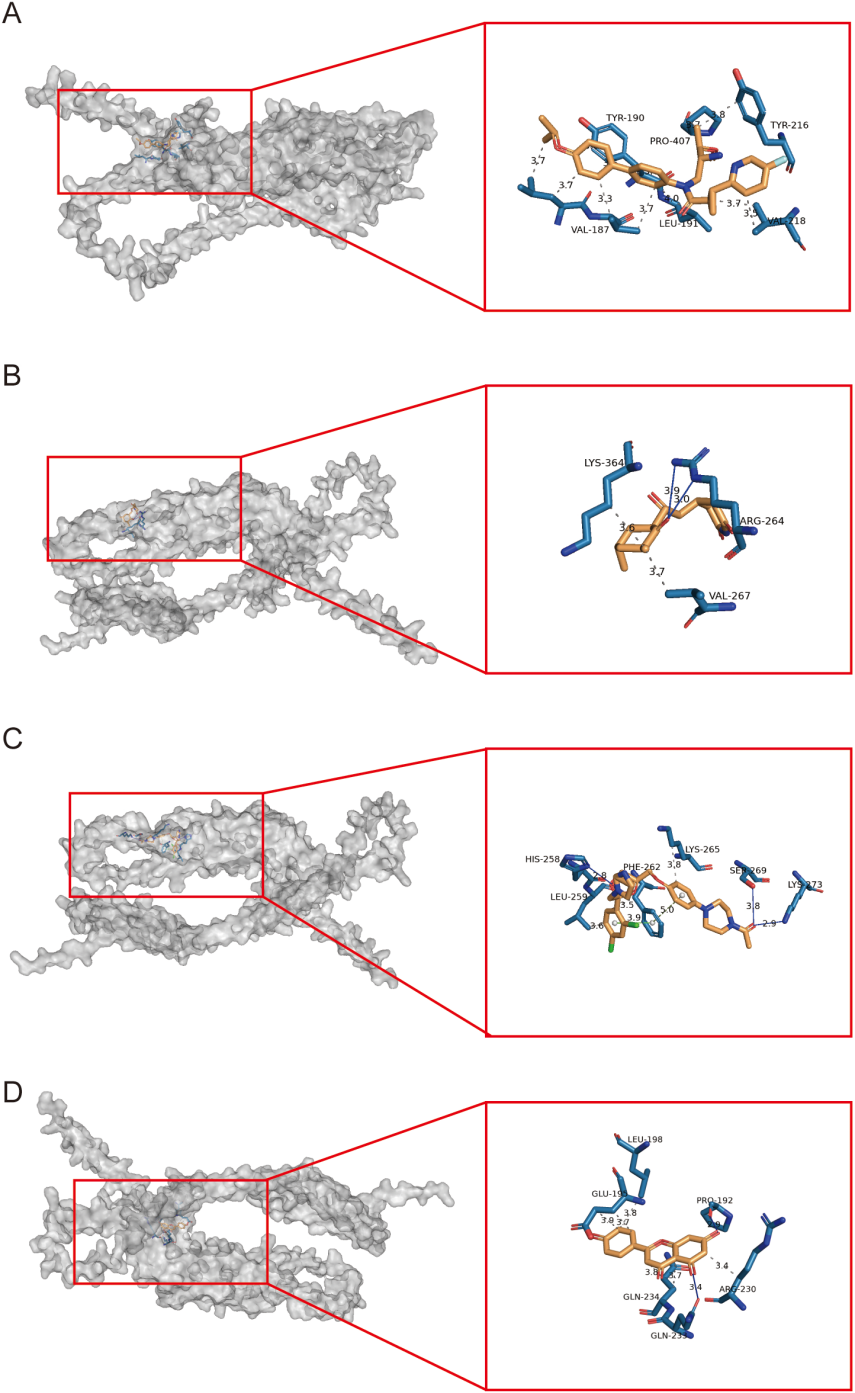
Molecular docking analysis of potential therapeutic compounds targeting PLIN2. **(A–D)** Molecular docking analyses of RTI-122, cycloheximide, ketoconazole, and apigenin with PLIN2.

Using an online drug prediction platform, we identified potential compounds targeting PLIN2, including RTI-122, cycloheximide, ketoconazole, and apigenin. Molecular docking analysis revealed favorable binding energies of -6.211, -6.303, -7.012, and -5.234 kcal/mol, respectively, all of which were below the -5 kcal/mol threshold, indicating stable binding interactions(**Figures 9A–D**). Molecular docking revealed distinct interaction patterns between PLIN2 and the four candidate drugs. RTI-122 primarily formed multiple hydrophobic interactions with residues such as LEU-191 and TYR-261. In contrast, cycloheximide established two hydrogen bonds with ARG-264. Ketoconazole engaged in a more diverse set of interactions, forming hydrogen bonds with LYS-273, SER-269, and HIS-258, alongside a Pi-Pi stacking interaction with PHE-262. Apigenin, however, primarily formed a hydrogen bond with GIM-233.

### 3.10 Experimental validation of the biomarker

**Figure 10:**
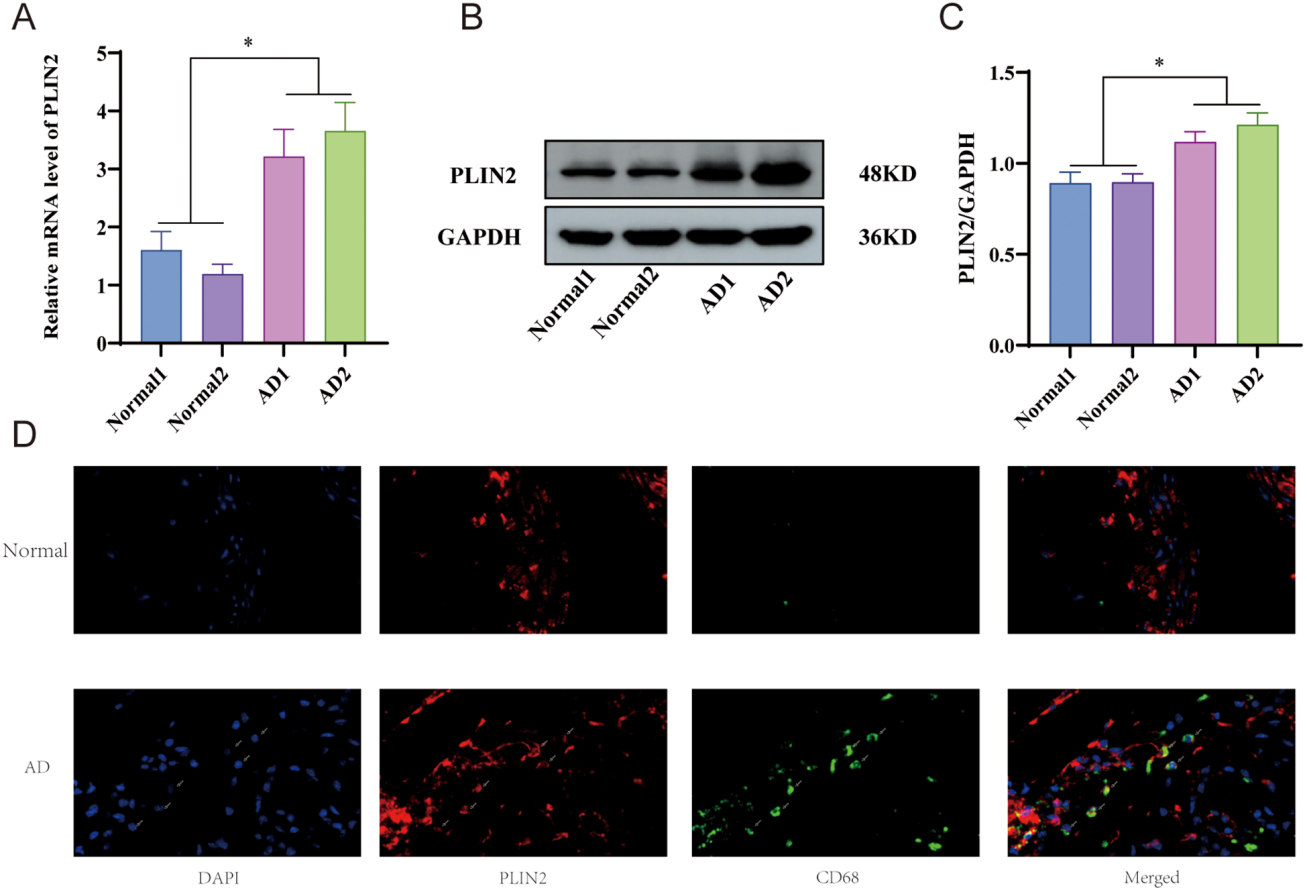
Experimental validation of the biomarker **(A)** qRT-PCR reveals the relative expression of PLIN2 mRNA. **(B, C)** Western blot analysis confirmed that PLIN2 protein levels were significantly elevated in aortic dissection tissues. **(D)** Immunofluorescence analysis revealed the staining patterns of DAPI (blue), PLIN2 (red), and CD68 (green), along with the merged channels, in both the normal control and aortic dissection groups. Small arrows indicate cells with evident co-localization of PLIN2 and CD68.

To validate the expression of the biomarker in aortic dissection, we performed quantitative PCR (qPCR) and Western blot analysis. The qPCR results revealed a significant increase in the relative mRNA level of PLIN2 in aortic dissection tissues (**Figure 10A**). Furthermore, Western blot analysis demonstrated that PLIN2 protein expression was also markedly upregulated (**Figures 10B, C**). The full, uncropped blot membrane has been included in **Supplementary Figure 8**.

To determine the cellular localization of PLIN2, immunofluorescence staining was conducted. The results showed co-localization of PLIN2 with CD68 (a macrophage marker) in dissected aortic tissues, indicating that PLIN2 is expressed in macrophages. In contrast, only basal expression of PLIN2 was detected in normal mouse aortic tissues, where CD68-positive cells were absent—suggesting a lack of macrophage infiltration—and no apparent co-localization was observed (**Figure 10D**).

## 4 Discussion

AD has long constituted a life-threatening cardiovascular emergency, posing substantial risks to both survival and functional outcomes. Notwithstanding progressive refinements in diagnostic and therapeutic approaches, the enduring burden of adverse outcomes underscores imperative needs for therapeutic innovation (38). While dysregulated lipid metabolism has been increasingly implicated in AD pathogenesis, the cell-type-specific heterogeneity of lipid metabolic reprogramming and its precise genetic regulators remain poorly characterized (9, 39). By combining scRNA-seq with supervised machine learning algorithms, we precisely mapped cell-type-specific lipid metabolism alterations in AD, revealing previously unrecognized vascular cell subsets exhibiting pathological metabolic profiles.

We identified PLIN2 and PLIN3 as previously unrecognized biomarkers linking macrophage function to lipid metabolism dysregulation in AD. Both genes show marked upregulation and strong association with perturbed lipid metabolic networks characteristic of AD progression. Through integrated analysis combining gene expression analyses, functional enrichment analyses and machine learning-based feature selection, we robustly identified and validated that these AD-associated genes are both statistically robust and mechanistically linked to AD pathology, offering new perspectives for understanding disease mechanisms and developing targeted interventions.

PLIN2 and PLIN3 are core lipid droplets (LDs)-associated proteins that orchestrate LD biogenesis and maturation. These proteins have been implicated in diverse pathological conditions including metabolic disorders (e.g., obesity, on-alcoholic fatty liver disease) and cellular processes such as autophagy, demonstrating their pleiotropic roles in cellular homeostasis (40). PLIN2, also referred to as differentiation-related protein (ADRP), is a member of the PAT family of lipid droplet-coating proteins that play fundamental roles in lipid storage and metabolism. Plin2 modulates the metabolic equilibrium between glucose and fatty acid utilization through its role in lipid droplet stabilization, which serves as a reservoir for excess fatty acids (41). Moreover, PLIN2 is capable of reducing the production of LDs or triglycerides by impeding the lipid droplet association of adipose triglyceride lipase (42). However, PLIN2 deficiency enhances lipolysis and accelerates fatty acid mobilization through upregulation of PPARγ signaling pathways, which concurrently modulate metabolism and amplify anti-inflammatory gene activation (41, 43). Similarly, in AD, we observed PLIN2 upregulation concomitant with PPARγ downregulation. This inverse correlation suggests PLIN2-mediated suppression of PPARγ signaling may contribute to AD pathogenesis, though further mechanistic studies are required to validate this hypothesis. Our study demonstrates for the first time that PLIN2 primarily regulates lipid metabolism in macrophages during AD. Other studies have also shown PLIN2’s important regulatory role in macrophage. For instance, in colorectal cancer PLIN2 promotes macrophage polarization towards the M2 phenotype and activates the CD36-dependent epithelial-mesenchymal transition (EMT) pathway in colorectal cancer cells, thereby enhancing tumor invasiveness (44). Otherwise, it is indicated that Perilipin 2-expressing mononuclear phagocytes exhibit significant retinal accumulation under diabetic conditions, where they promote microvascular degeneration through PPARγ signaling activation (45). Therefore, PLIN2 could represent a potential therapeutic target, as suppression of its activity may attenuate AD-associated inflammation and improve clinical outcomes.

PLIN3, also called TIP47, is widely expressed across various tissues. It attaches to newly formed lipid droplets and can dynamically associate with or dissociate from LDs while remaining stable in the cytosol (46, 47). In macrophage, it is proposed that TIP47 may act as a carrier protein for free fatty acids and in this way participates in conversion of Uptake of lipids by macrophages (MPhi) into foam cells (48). The mechanisms of TIP47 in various diseases remain unclear. Although TIP47 expression is elevated in macrophages during aortic dissection, its specific pathological mechanisms require further investigation.

An additional intriguing observation is that PLIN2 and PLIN3 both are related to autophagy which is a pathway that intersects with lipid homeostasis (49). Consistently, our GSEA analysis of up-regulated DEGs between PLIN2-high and PLIN2-low groups confirms these mechanistic insights (**Figure 6**). Autophagy is a lysosome-dependent catabolic process that degrades proteins, lipids, and organelles to maintain cellular homeostasis (50). Enrichment analysis of the 26 machine learning-selected genes revealed multiple lysosome-related pathways, further supporting this conclusion. With respect to the underlying mechanisms, Susmita Kaushik reported that the CMA-dependent degradation of PLIN2 and PLIN3 serves as a prerequisite for Adipose triglyceride lipase (ATGL) to bind lipid droplets and initiate lipolysis (49). In other organs, PLIN2 also modulates the catabolism of cytosolic lipid droplets (LDs) to maintain cellular lipid homeostasis (51, 52). However, the mechanistic involvement of autophagy in macrophages within aortic dissection has not yet been elucidated. Modulation of macrophage autophagy in aortic dissection may represent a potential therapeutic strategy, though further experimental validation is required.

### 4.1 Limitation

Although this study offers novel insights, several limitations should be acknowledged. First, our study relied on the integration of three independent single-cell RNA-seq datasets to achieve a sufficient cell number for robust analysis. We acknowledge that this integrative approach, despite rigorous correction using advanced batch-effect removal algorithms (e.g., Harmony), may inherently introduce residual technical variation that could confound the biological signals. This represents a common trade-off in computational biology between sample size and data homogeneity. Future studies with larger, prospectively collected cohorts will be invaluable to corroborate our findings in a more uniform analytical framework. Next, while we established a correlation between lipid metabolic activity and PLIN2/PLIN3 expression, the exact molecular mechanisms by which these genes modulate lipid metabolism and contribute to AD pathogenesis require further elucidation. Moreover, our study focuses predominantly on transcriptomic alterations within the AD landscape, which does not explore post-transcriptional modifications and protein-level changes, all of which significantly contribute to disease pathology. Furthermore, both animal model experiments and clinical studies will be essential to validate their diagnostic and therapeutic potential in preclinical and human contexts. Although these limitations exist, our study advances the understanding of AD molecular mechanisms and proposes potential diagnostic markers and intervention strategies. Finally, regarding the drug candidates targeting PLIN2, our validation relied solely on molecular docking. While this computational approach successfully established PLIN2 as a tractable target with stable ligand binding, it is inherently preliminary. Future work necessitates more advanced simulations, such as molecular dynamics, and crucially, experimental validation to confirm the functional efficacy of these interactions.

## 5 Conclusion

Our investigation provides the first single-cell resolution analysis of lipid metabolic heterogeneity in aortic dissection, demonstrating macrophages as pivotal mediators of inflammatory responses. The discovery of PLIN2 and PLIN3 as central modulators of lipid metabolism in aortic dissection highlights their potential as both diagnostic biomarkers and intervention targets.

## Data Availability

The original contributions presented in the study are included in the article/**Supplementary Material**. Further inquiries can be directed to the corresponding author.
