# Correction: Identification of novel lipid metabolism-related biomarkers of aortic dissection by integrating single-cell RNA sequencing analysis and machine learning algorithms

**DOI:** 10.3389/fimmu.2025.1742065

**Published:** 2025-11-20

**Authors:** Zhechen Li, Yusong Deng, Fei Xiao, Jiashu Sun, Qixu Zhao, Zetong Zheng, Gang Li

**Affiliations:** 1 Beijing Luhe Hospital, Capital Medical University, Beijing, China; 2 Pediatric Cardiac Center, Beijing Anzhen Hospital, Capital Medical University, Beijing, China; 3 Beijing Institute of Heart Lung and Blood Vessel Diseases, Beijing, China; 4 Department of Anesthesiology, Beijing Anzhen Hospital, Capital Medical University, Beijing, China

**Keywords:** macrophage, lipid metabolism, aortic dissection, PLIN2, single-cellRNA sequencing

An incorrect grant number was provided for “Beijing Natural Science Foundation”. The correct number is “L258027”.

The original version of this article has been updated.

