# Supplementary material for: Identification of novel lipid metabolism-related biomarkers of aortic dissection by integrating single-cell RNA sequencing analysis and machine learning algorithms
Source: Front Immunol. 2025 Oct 30;16:1681989. doi: 10.3389/fimmu.2025.1681989 (PMC12611683; doi:10.3389/fimmu.2025.1681989)
Supplement: Supplementary file 1 [file DataSheet1.zip › Supplementary Figures' legends/Supplementary Figures.docx]

Supplementary Figures


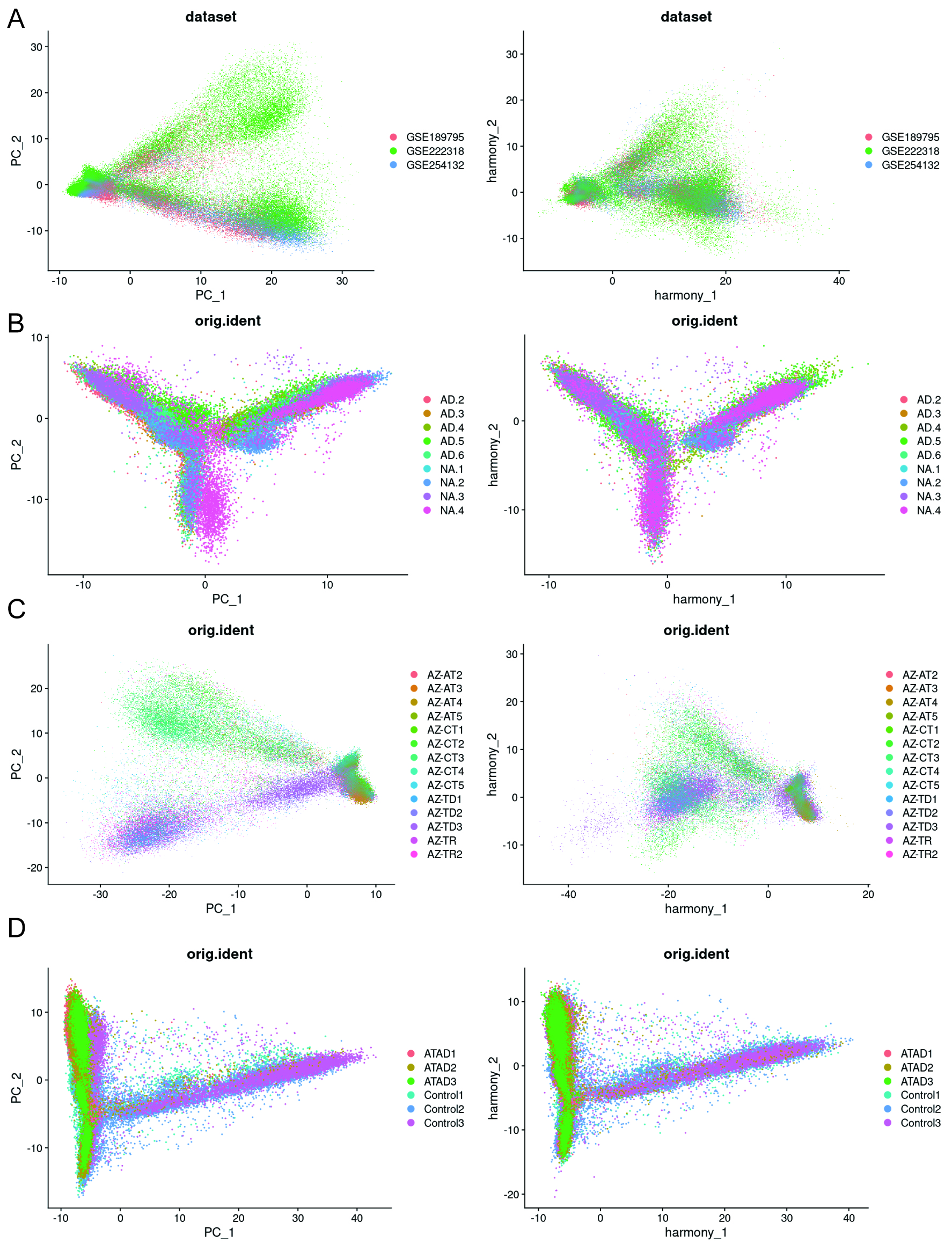


**Supplementary Figure 1.**(A-D)**PCA plot demonstrating batch effect correction by Harmony in merged scRNA-seq,GSE189795,GSE222318 and GSE254132 respectively.** The comparison of PCA before and after applying the Harmony algorithm reveals a substantial reduction in batch effects.


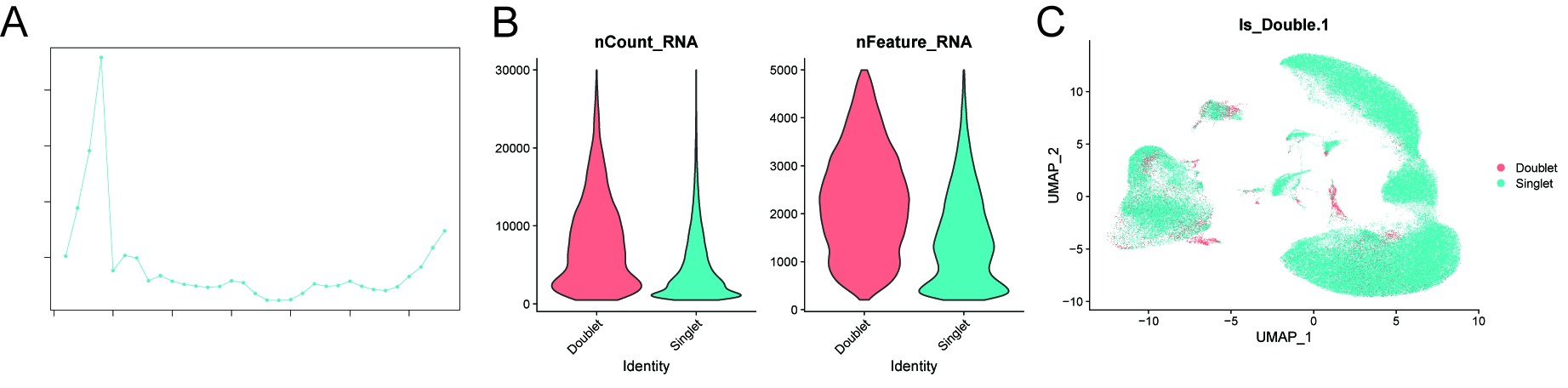


**Supplementary Figure 2.**(A)**The determination the optimal pK value.(B)The violin plots show the difference of nCount_RNA and nFeature_RNA between potential doublet and singlet.(C)The UMAP** visualization **revealed a heterogeneous distribution pattern of potential doublet and singlet.**


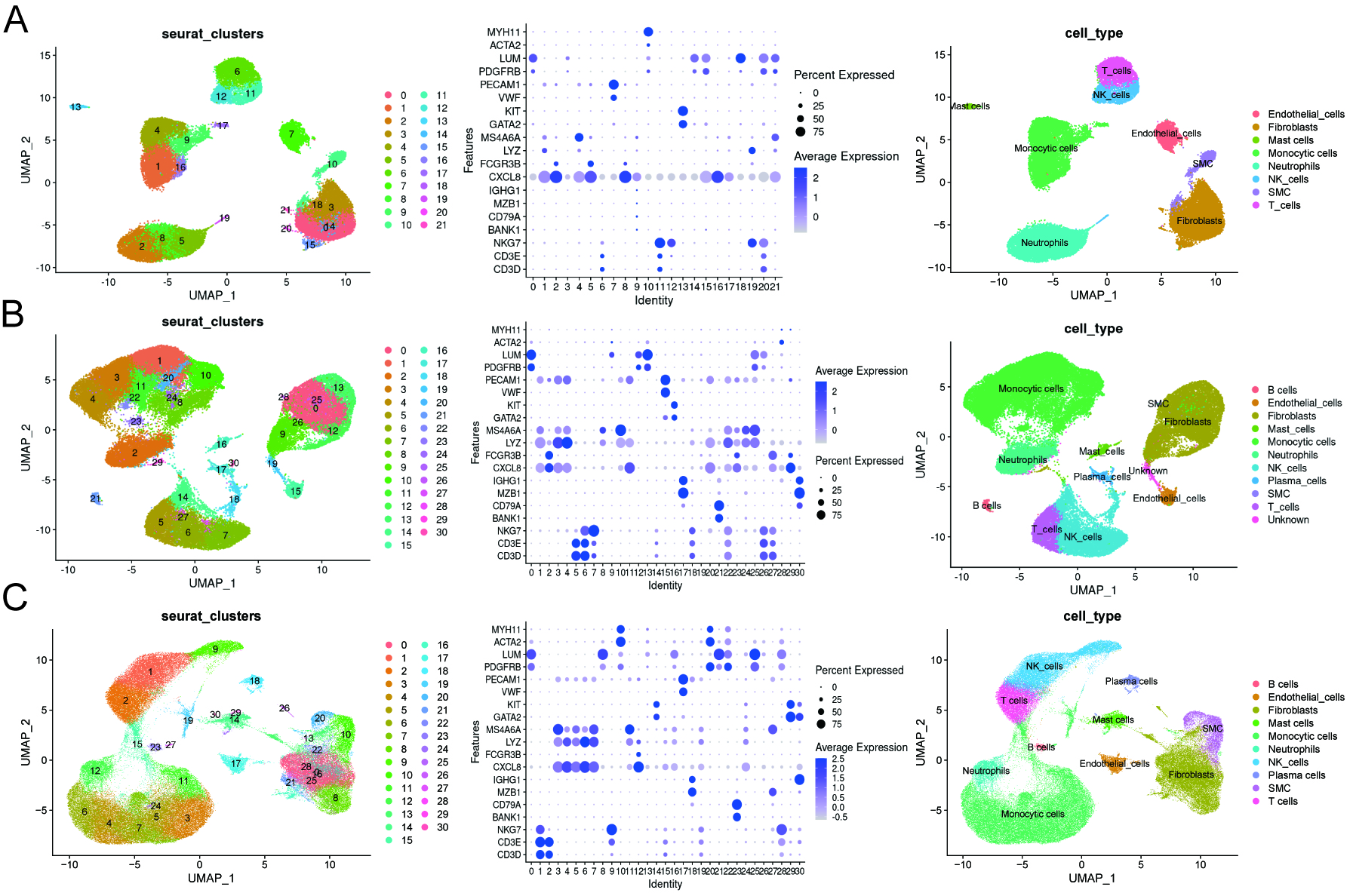


**Supplementary Figure 3.**(A-C) Panels from top to bottom correspond to datasets GSE254132, GSE189795, and GSE222318, respectively. For each dataset, the left UMAP plot shows the unsupervised clustering of cells; the middle dot plot displays the expression distribution of key marker genes across these clusters; and the right UMAP plot presents the final cell type annotation based on these markers.


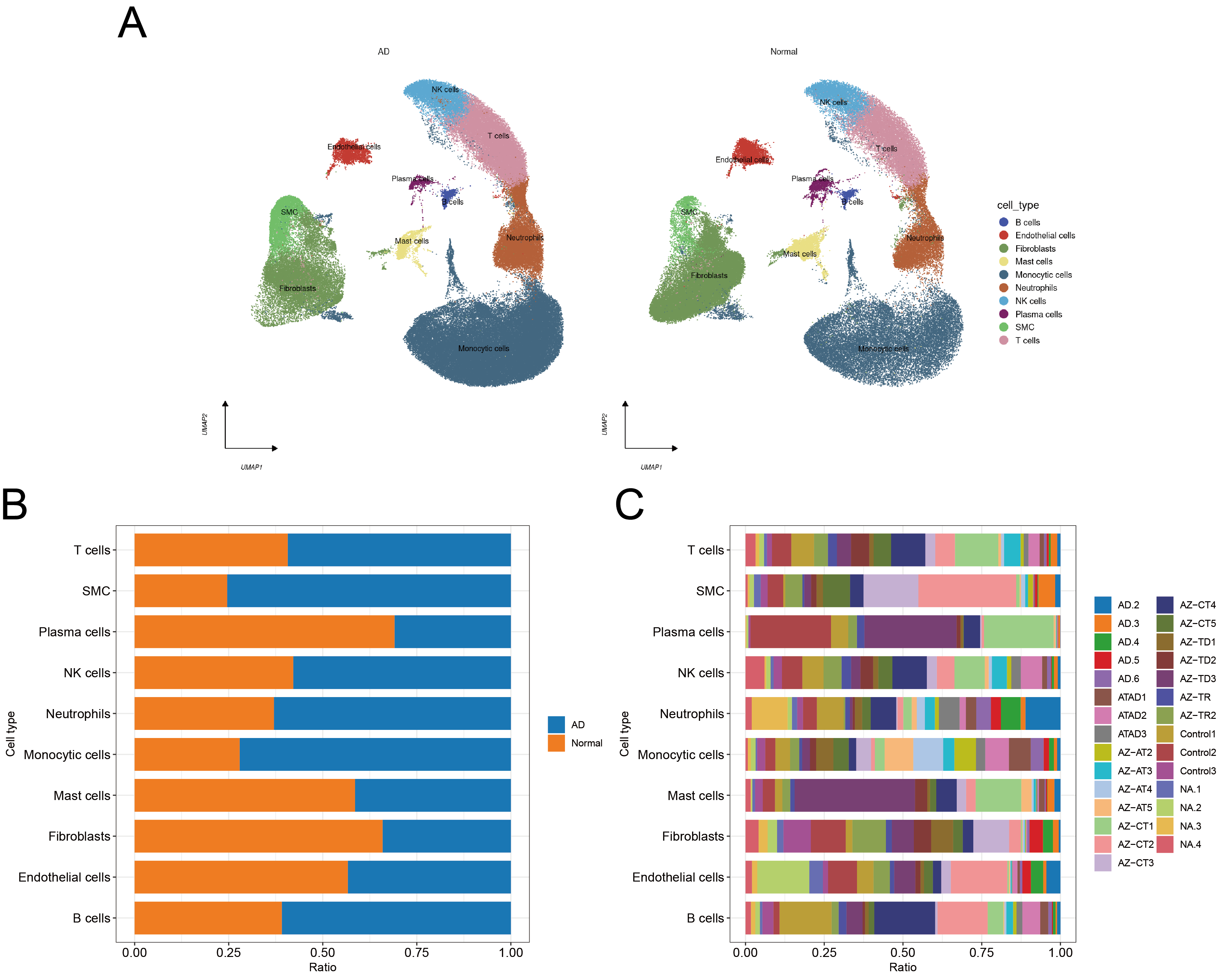


**Supplementary Figure 4.** (A)The UMAP plot illustrates the distribution patterns of diverse cell populations between the AD group and the normal group.(B)The diagram shows cell type proportion comparison between AD group and normal group. (C) Stacked bar plot illustrating the relative abundances of all identified cell types in 29 samples.


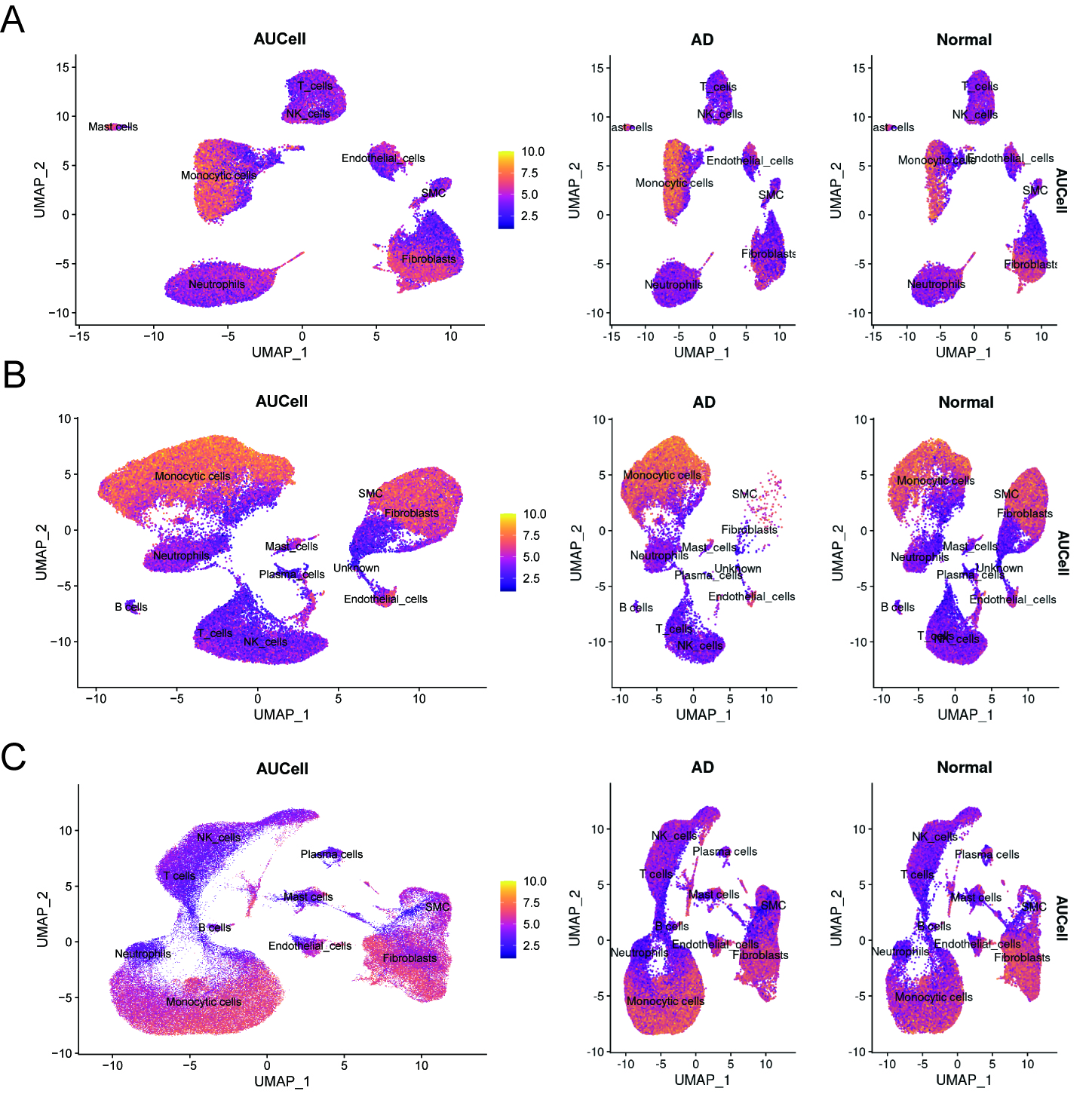


**Supplementary Figure 5.** (A-C) Panels from top to bottom correspond to datasets GSE254132, GSE189795, and GSE222318.For each dataset, the left UMAP visualization illustrates the overall distribution of lipid metabolism scores across all cells. The right UMAP plot highlights differences in lipid metabolism score distribution between the AD and normal groups.


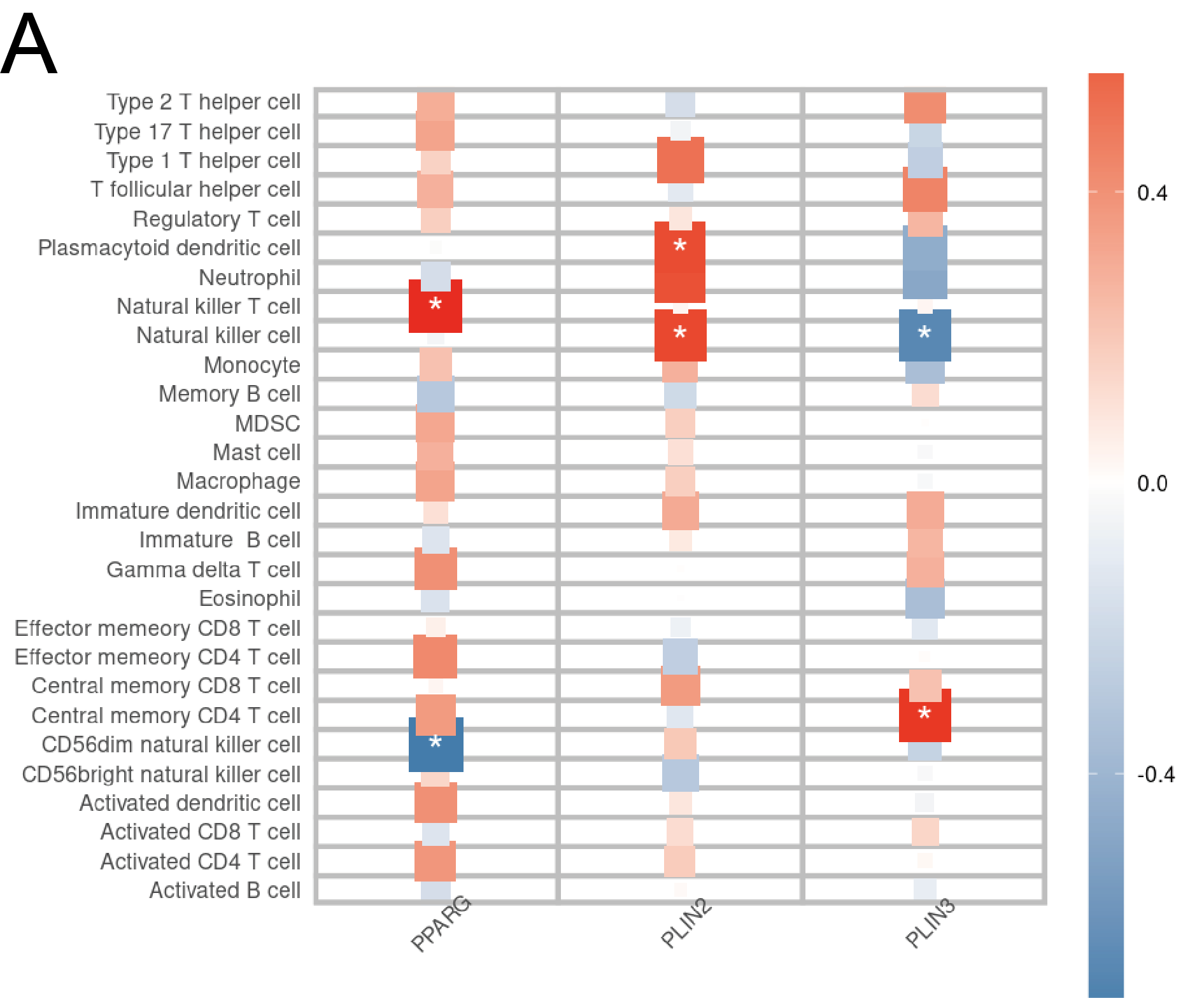


**Supplementary Figure 6.** (A) Immune infiltration analysis illustrates the correlation between the biomarkers and immune cell populations.


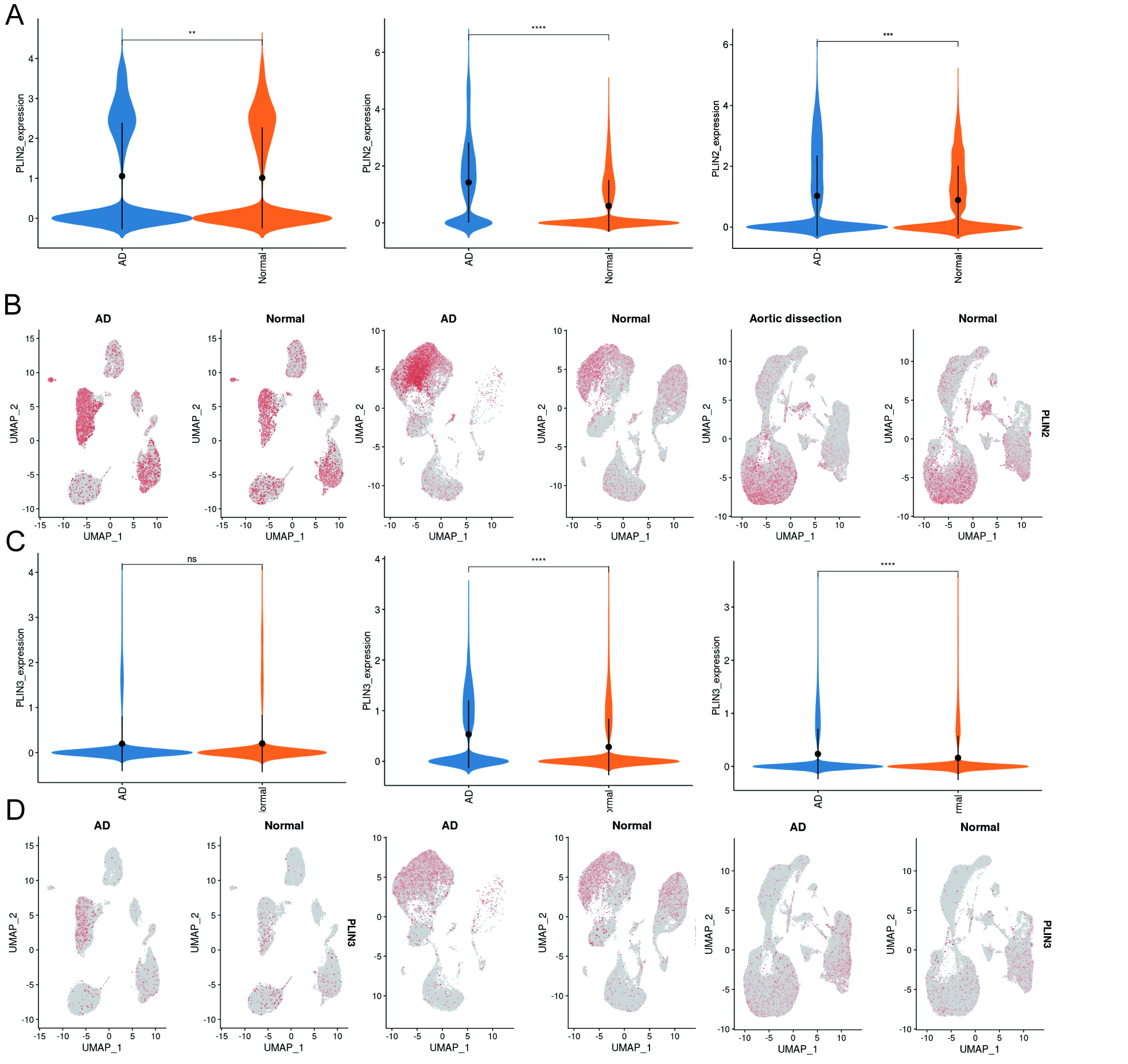


**Supplementary Figure 7.**(A-B)Expression patterns of PLIN2 in datasets GSE254132 (left), GSE189795 (middle), and GSE222318 (right), respectively. PLIN2 shows a significant increase in the AD group compared to the normal group in all independent cohorts.(C-D)Expression patterns of PLIN2 in datasets GSE254132 (left), GSE189795 (middle), and GSE222318 (right), respectively.PLIN2 shows a significant increase in the AD group compared to the normal group in all independent cohorts expect the GSE254132.


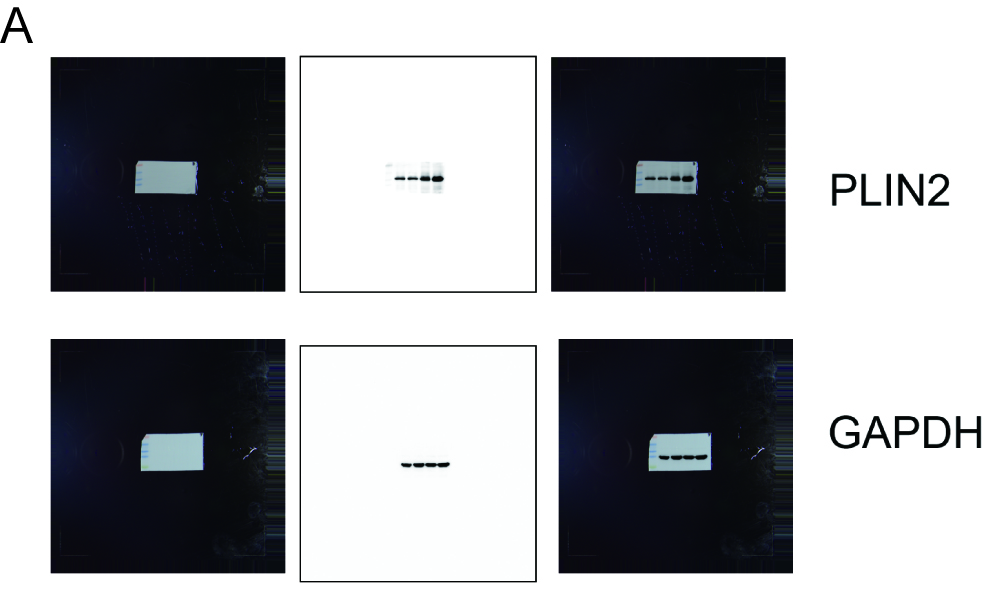


**Supplementary Figure 8.**(A)The full, uncropped blot membrane of western blotting.
