# Supplementary figures and images for: Identification of novel lipid metabolism-related biomarkers of aortic dissection by integrating single-cell RNA sequencing analysis and machine learning algorithms
Source: Front Immunol. 2025 Oct 30;16:1681989. doi: 10.3389/fimmu.2025.1681989 (PMC12611683; doi:10.3389/fimmu.2025.1681989)

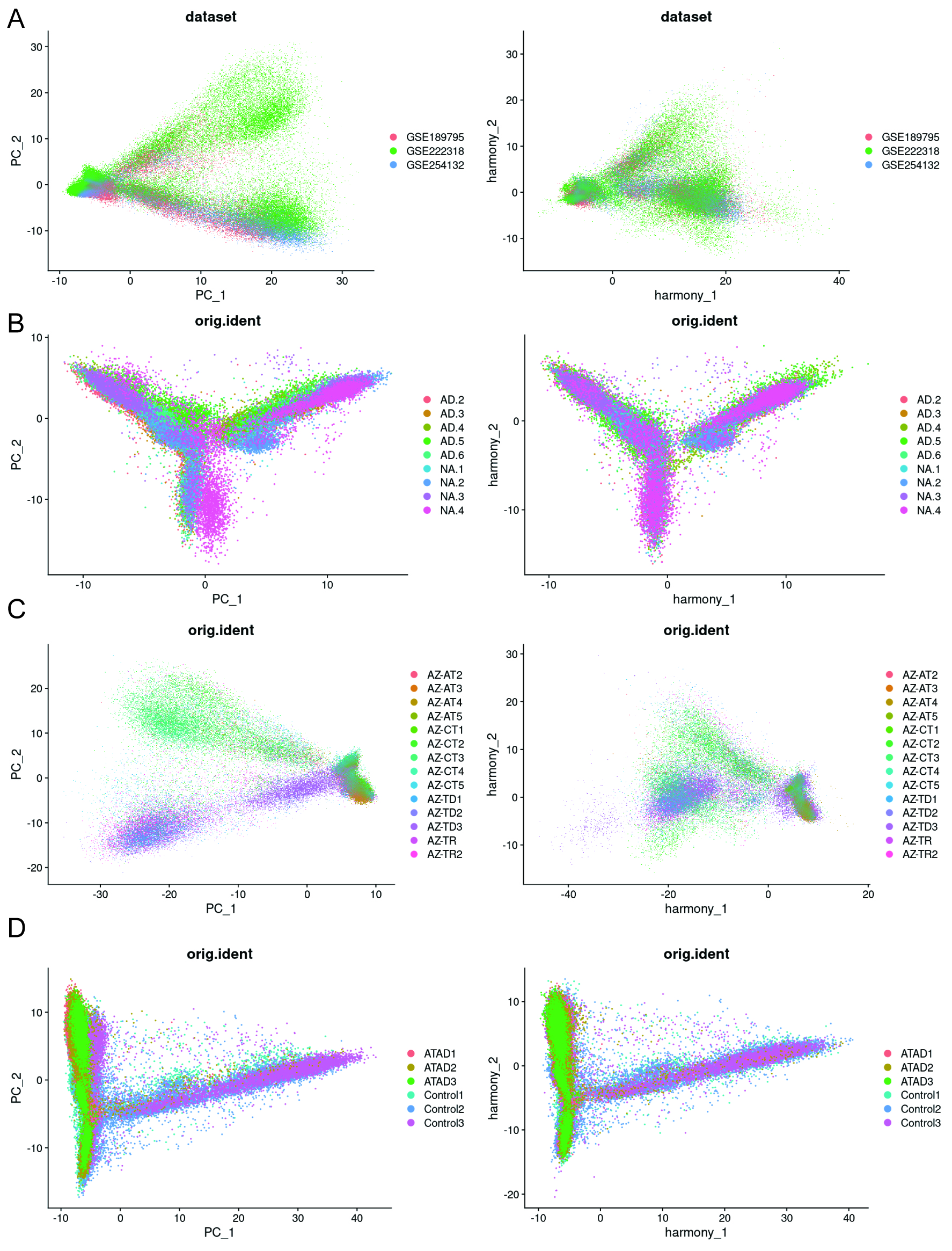

Supplement: Supplementary file 1 [file DataSheet1.zip › Supplementary Figures1-8/Supplementary Figure1.jpg]

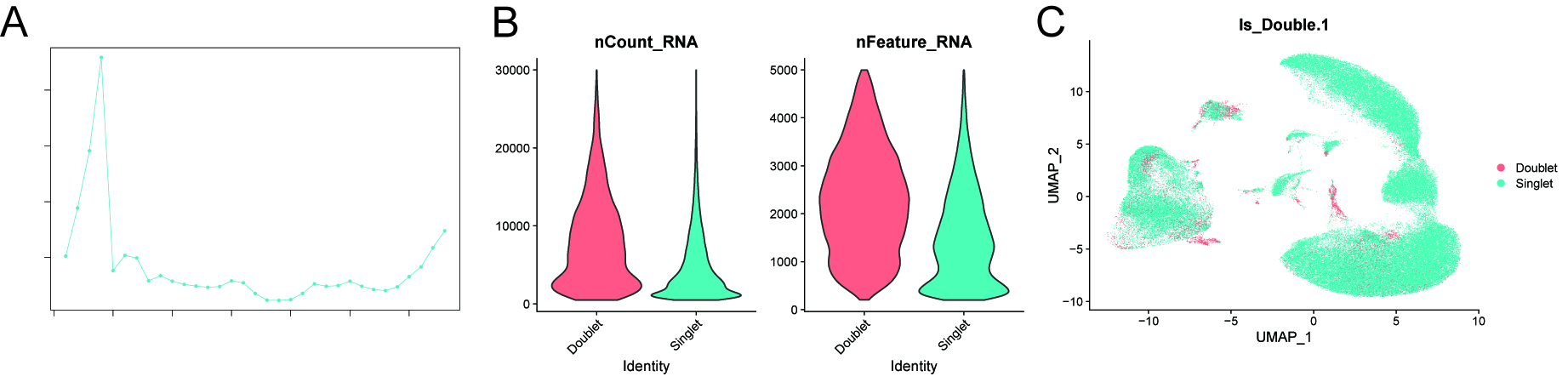

Supplement: Supplementary file 1 [file DataSheet1.zip › Supplementary Figures1-8/Supplementary Figure2.jpg]

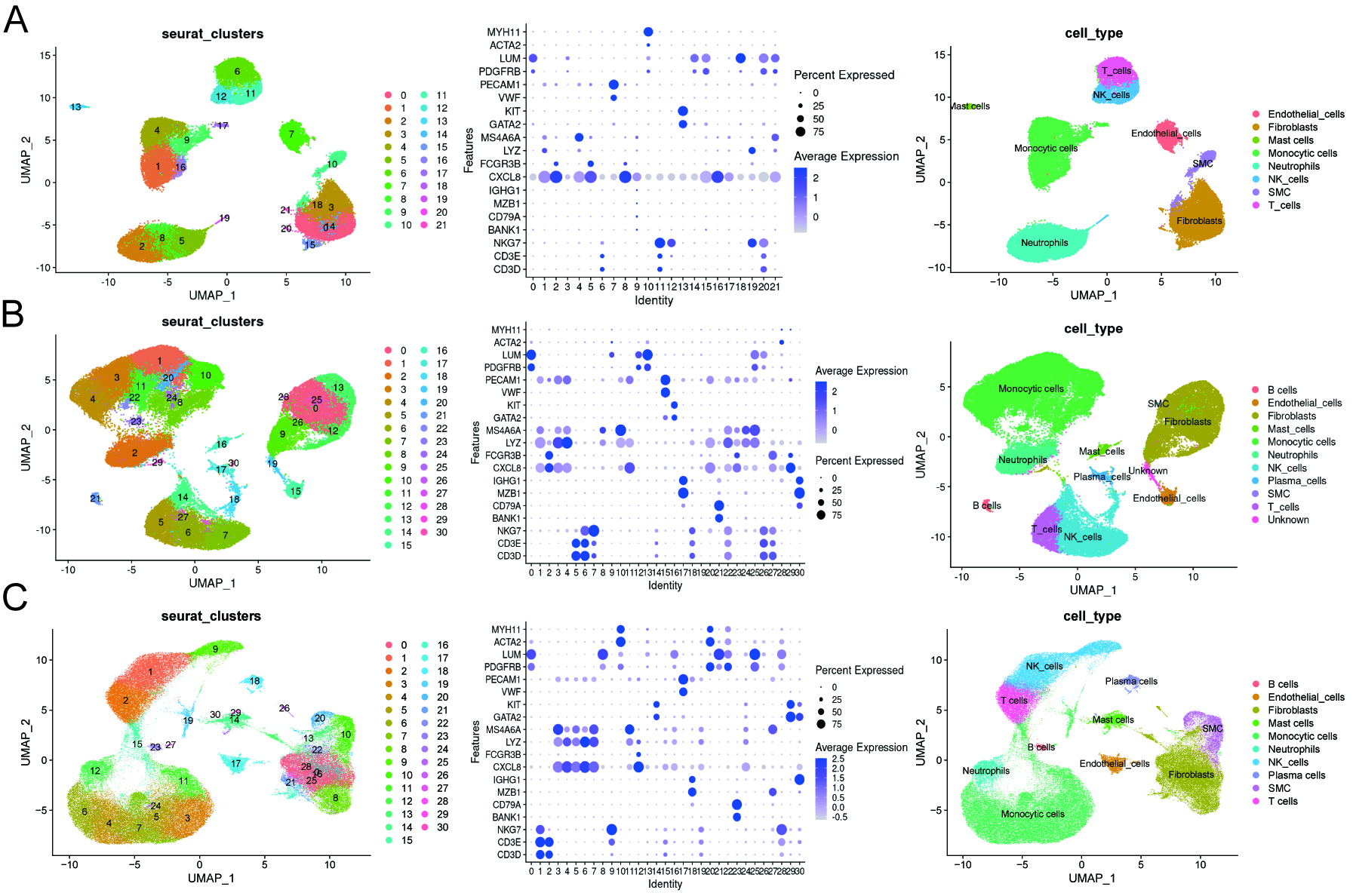

Supplement: Supplementary file 1 [file DataSheet1.zip › Supplementary Figures1-8/Supplementary Figure3.jpg]

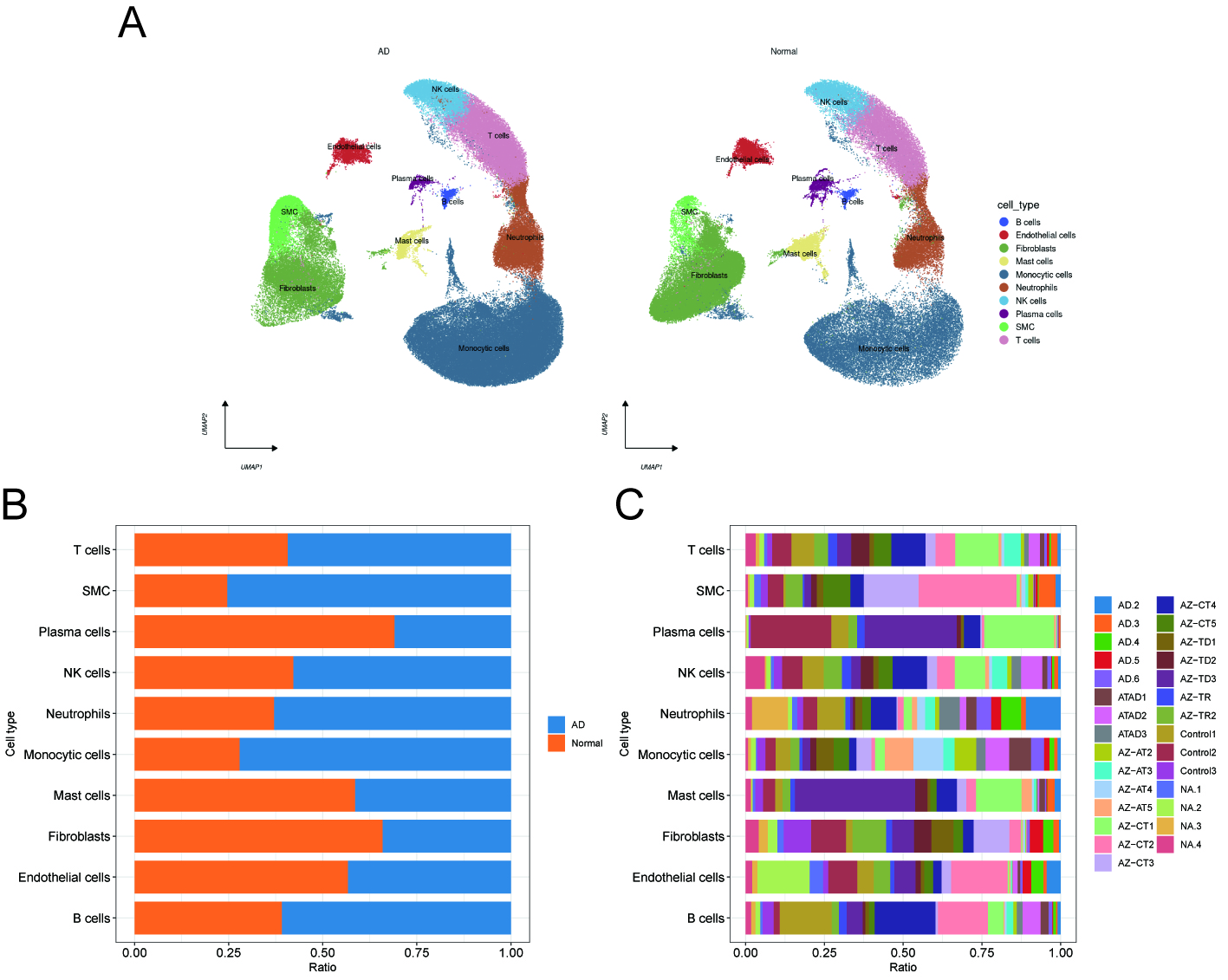

Supplement: Supplementary file 1 [file DataSheet1.zip › Supplementary Figures1-8/Supplementary Figure4.jpg]

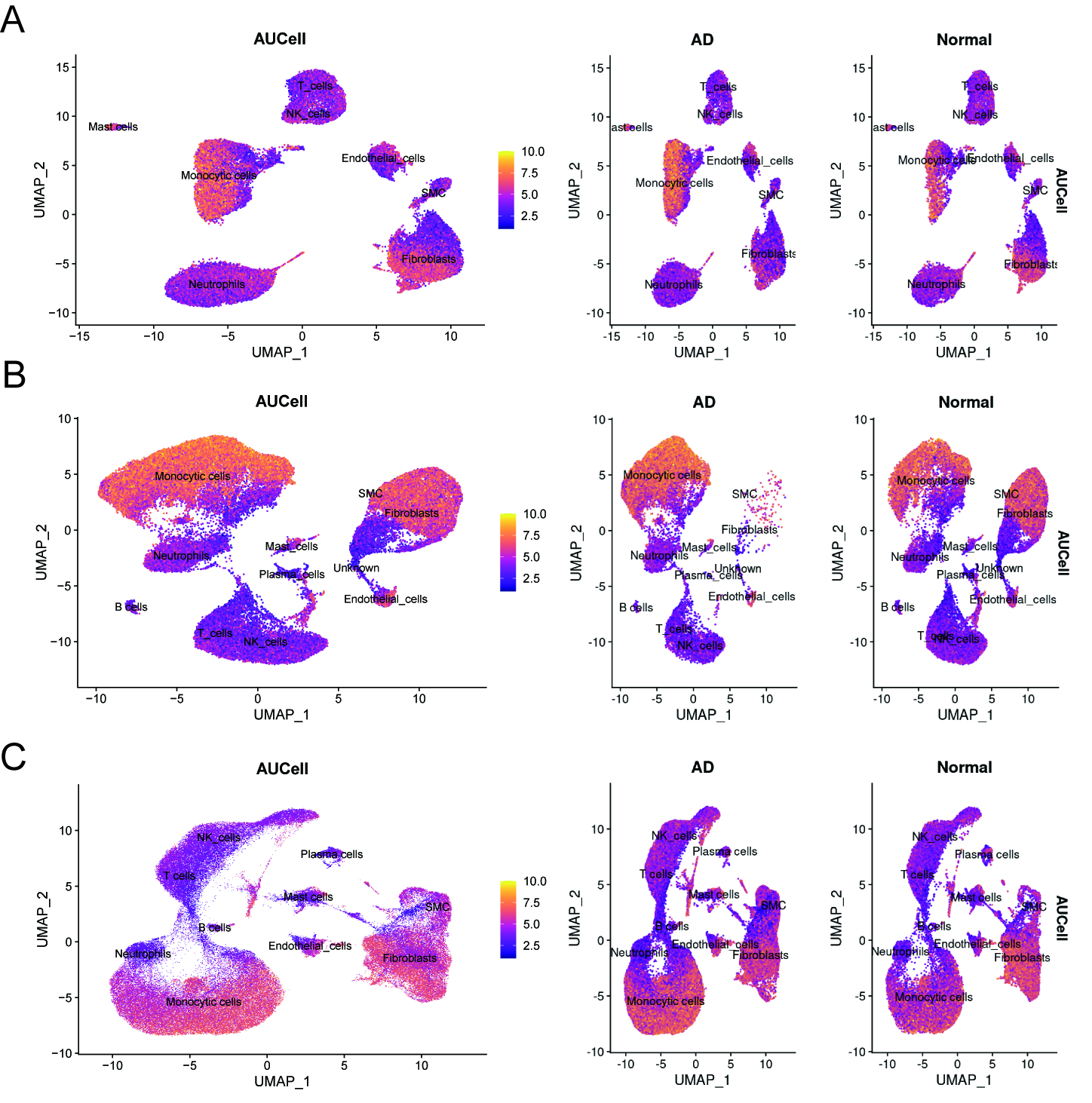

Supplement: Supplementary file 1 [file DataSheet1.zip › Supplementary Figures1-8/Supplementary Figure5.jpg]

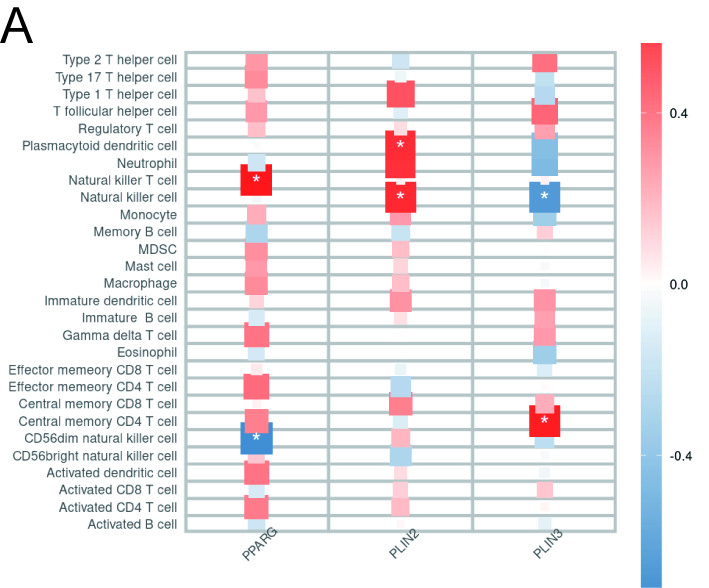

Supplement: Supplementary file 1 [file DataSheet1.zip › Supplementary Figures1-8/Supplementary Figure6.jpg]

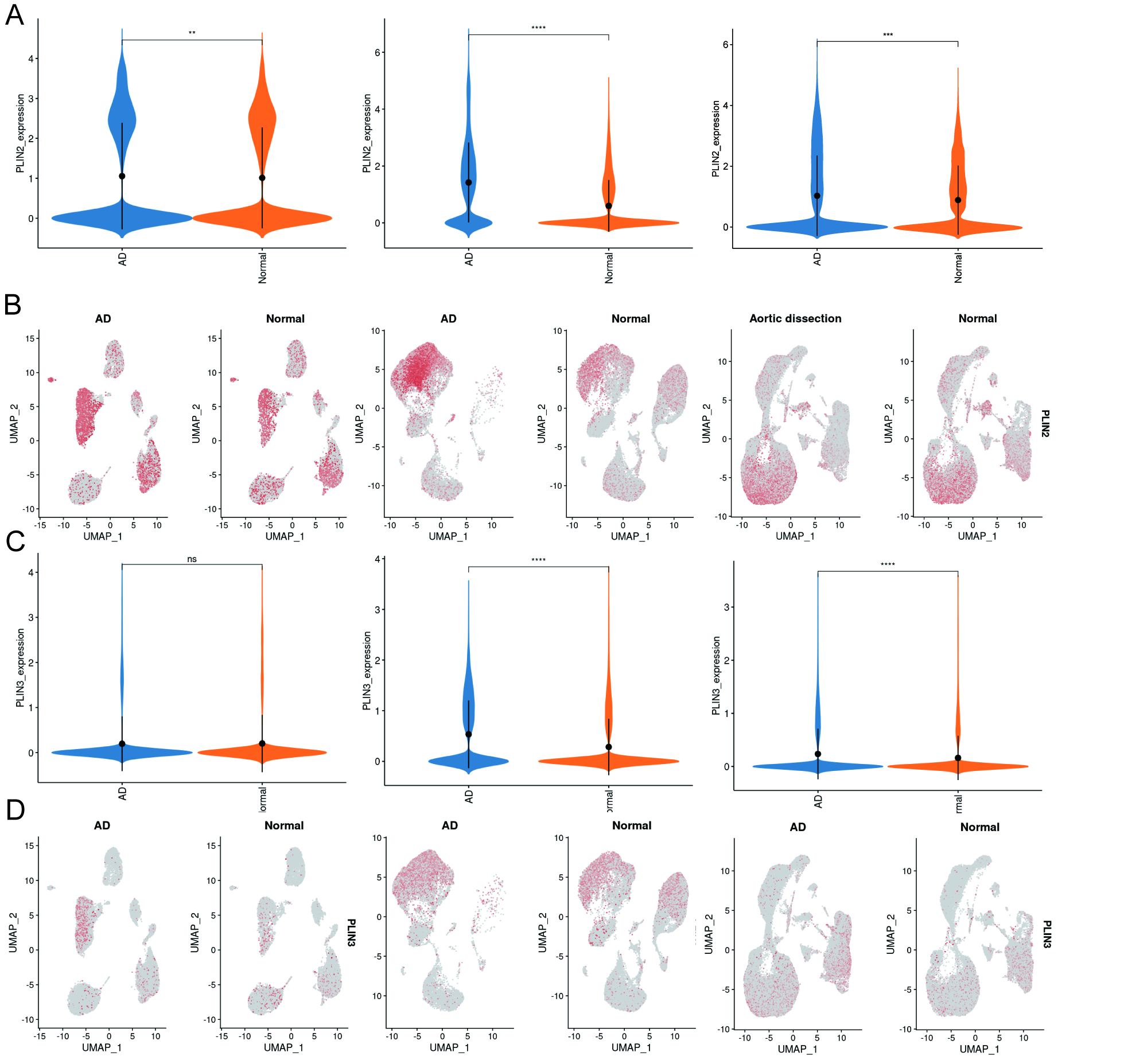

Supplement: Supplementary file 1 [file DataSheet1.zip › Supplementary Figures1-8/Supplementary Figure7.jpg]

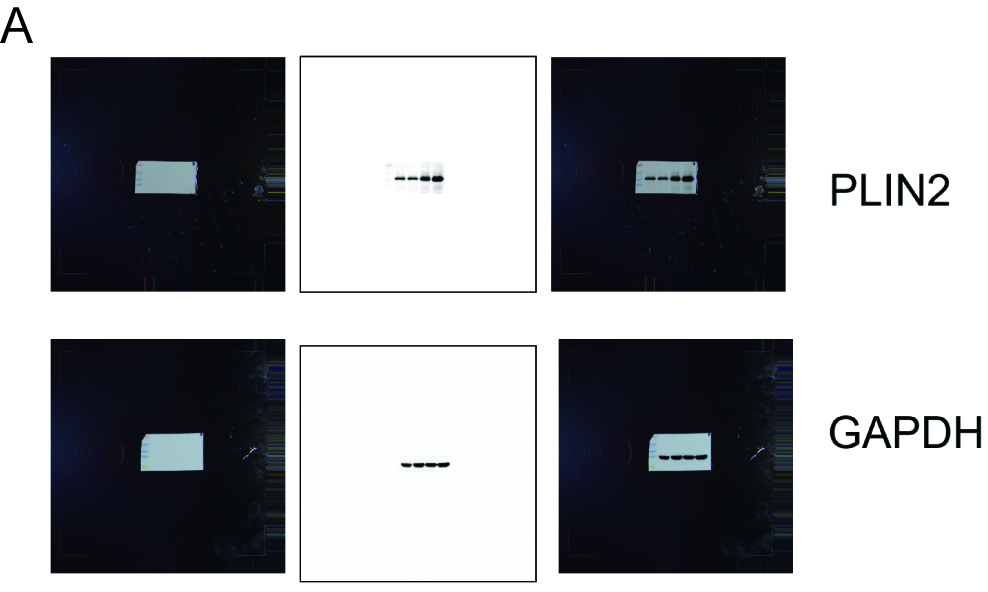

Supplement: Supplementary file 1 [file DataSheet1.zip › Supplementary Figures1-8/Supplementary Figure8.jpg]
